# Activation of temperature-sensitive TRPV1-like receptors in ARC POMC neurons reduces food intake

**DOI:** 10.1371/journal.pbio.2004399

**Published:** 2018-04-24

**Authors:** Jae Hoon Jeong, Dong Kun Lee, Shun-Mei Liu, Streamson C. Chua, Gary J. Schwartz, Young-Hwan Jo

**Affiliations:** 1 Division of Endocrinology, Department of Medicine, Albert Einstein College of Medicine, New York, United States of America; 2 Department of Physiology, College of Medicine, Gyeongsang National University, Jinju, Korea; 3 Department of Molecular Pharmacology, Albert Einstein College of Medicine, New York, United States of America; McGill Centre for Research in Neuroscience, Canada

## Abstract

Proopiomelanocortin (POMC) neurons in the arcuate nucleus of the hypothalamus (ARC) respond to numerous hormonal and neural signals, resulting in changes in food intake. Here, we demonstrate that ARC POMC neurons express capsaicin-sensitive transient receptor potential vanilloid 1 receptor (TRPV1)-like receptors. To show expression of TRPV1-like receptors in ARC POMC neurons, we use single-cell reverse transcription-polymerase chain reaction (RT-PCR), immunohistochemistry, electrophysiology, TRPV1 knock-out (KO), and TRPV1-Cre knock-in mice. A small elevation of temperature in the physiological range is enough to depolarize ARC POMC neurons. This depolarization is blocked by the TRPV1 receptor antagonist and by *Trpv1* gene knockdown. Capsaicin-induced activation reduces food intake that is abolished by a melanocortin receptor antagonist. To selectively stimulate TRPV1-like receptor-expressing ARC POMC neurons in the ARC, we generate an adeno-associated virus serotype 5 (AAV5) carrying a Cre-dependent channelrhodopsin-2 (ChR2)–enhanced yellow fluorescent protein (eYFP) expression cassette under the control of the two neuronal POMC enhancers (nPEs). Optogenetic stimulation of TRPV1-like receptor-expressing POMC neurons decreases food intake. Hypothalamic temperature is rapidly elevated and reaches to approximately 39 °C during treadmill running. This elevation is associated with a reduction in food intake. Knockdown of the *Trpv1* gene exclusively in ARC POMC neurons blocks the feeding inhibition produced by increased hypothalamic temperature. Taken together, our findings identify a melanocortinergic circuit that links acute elevations in hypothalamic temperature with acute reductions in food intake.

## Author summary

Intense exercise acutely decreases appetite and subsequent food intake. As exercise is accompanied by increased body temperature, we hypothesized that a rise in body temperature during exercise plays a role in reducing food intake. The hypothalamic neurons are major components of the neural circuits that control feeding in response to hormones and neural signals. Among hypothalamic neurons, those that express proopiomelanocortin (POMC) in the arcuate nucleus of the hypothalamus are important in controlling food intake. In this study, we found that these POMC-expressing neurons express TRPV1-like thermoreceptors that are activated by an increase in temperature within the physiological range in mice. We also showed that an increase in body temperature during exercise is directly sensed by these POMC-expressing neurons through activation of the TRPV1-like receptors. Hence, this study provides a novel perspective on the cellular mechanisms underlying energy balance: body temperature reduces food intake via TRPV1-like receptors in POMC-expressing neurons in the arcuate nucleus of the hypothalamus.

## Introduction

Body temperature changes according to the circadian rhythm, physical activities, and external factors such as food intake [1–3]. Although it is expected that a rise in body temperature is rapidly conveyed to the hypothalamus via the blood stream, the physiological consequences of such an increase in hypothalamic temperature have not been studied. The transient receptor potential vanilloid subfamily members 1–4 (TRPV1, 2, 3, and 4) in peripheral sensory neurons play a role in accurately sensing ambient temperature and in adapting to environmental temperature changes through behavioral and physiological responses [4–9]. In the early 1960s, Nakayama and colleagues showed that local hypothalamic heating from 36.7 °C to 38.7 °C increases hypothalamic neuronal activity [10], suggesting that, like peripheral sensory neurons, hypothalamic neurons are able to respond to changes in temperature, possibly via activation of temperature-sensitive transient receptor potential vanilloid (TRPV) channels. Indeed, neurophysiological, genetic, and in situ hybridization studies have described expression of TRPV receptors, in particular TRPV1 channels, specifically in hypothalamic neurons [11–14].

However, the presence of functional TRPV1 in the hypothalamus is still controversial. In fact, TRPV1 reporter mouse studies have shown very limited expression of TRPV1 receptors in the hypothalamus [12]. Second, TRPV1-expressing vasopressin neurons show temperature-sensitive inward currents at temperatures above 35 °C [13,15,16], which is in contrast to the prior findings that recombinant TRPV1 receptors are activated only by noxious temperatures (>42 °C) [4]. Third, transcriptome analysis of agouti-related peptide (AgRP) and proopiomelanocortin (POMC) neurons in the arcuate nucleus of the hypothalamus (ARC) shows no expression of *Trpv1* transcripts [17]. Interestingly, some ARC POMC neurons are situated outside the blood–brain barrier [18,19], which permits them to detect acute changes in temperature. Therefore, we sought to determine whether ARC POMC neurons express temperature-sensitive TRPV1 receptors.

Using multiple independent techniques, we found that ARC POMC neurons expressed capsaicin- and temperature-sensitive TRPV1 subunit-containing receptors (TRPV1-like). Pharmacologic and optogenetic activation of ARC POMC neurons that express TRPV1-like channels reduced food intake. Increased body temperature during exercise was transmitted to the ARC. This elevation was associated with a reduction in food intake that was blocked by a melanocortin receptor antagonist. These results support the interpretation that activation of capsaicin-sensitive TRPV1-like receptors in ARC POMC neurons contributes to the regulation of acute food intake.

## Results

### ARC POMC neurons express capsaicin-sensitive TRPV1-like receptors

The ARC expresses *Trpv1* mRNA and [^3^H]resiniferatoxin(RTX) binding sites [11,14]. Among the class of TRPV channels, only TRPV1 channels respond to capsaicin [4,20–23]. Consequently, we examined whether ARC POMC neurons respond to the selective TRPV1 receptor agonist capsaicin [24–26]. Membrane potentials were recorded from ARC POMC–enhanced green fluorescent protein (eGFP) neurons in hypothalamic slices from POMC-eGFP mice in the presence of synaptic blockers (6-cyano-7-nitroquinoxaline-2,3-dione [CNQX], 10 μM, and picrotoxin, 100 μM). Treatment with capsaicin (100 nM) significantly depolarized and triggered action potentials in ARC POMC neurons (S1A and S1B Fig). In addition, treatment with another potent and selective TRPV1 receptor agonist RTX [27] (100 nM) also depolarized ARC POMC, but not neuropeptide Y (NPY) neurons (S1C, S1D, S5B and S5C Figs). Capsaicin-induced depolarization was completely blocked by treatment with the TRPV1 receptor antagonist AMG9810 (10 μM) [28] (S1E and S1F Fig). In addition, the TRPV1 receptor-induced depolarization was still present in the presence of tetrodotoxin (TTX) (S1G and S1H Fig). These pharmacological results support the existence of functional capsaicin-sensitive TRPV1 channels expressed by ARC POMC neurons.

**Fig 1:**
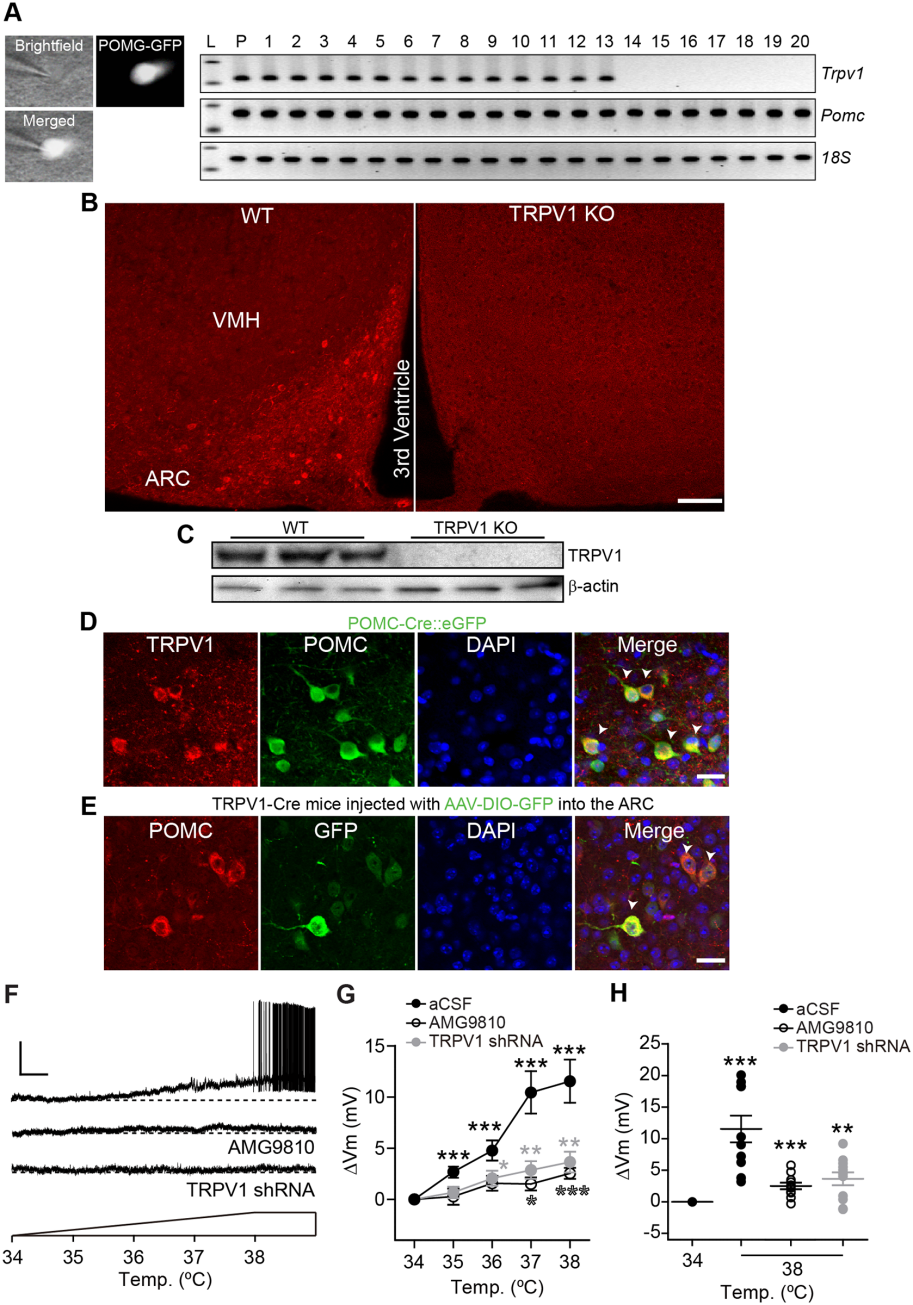
ARC POMC neurons express temperature-sensitive TRPV1 receptors. **(A)** Single-cell RT-PCR analysis of POMC neurons showing *Trpv1* mRNA expression. Images showing POMC-eGFP neurons in hypothalamic slices (left). Right: Transcripts were collected from 20 different POMC neurons (lane, 1–20). L: ladder (*Trpv1*, 120 bp; *Pomc*, 170 bp; *18S rRNA*, 121 bp). P: positive control. **(B)** Images of fluorescence confocal microscopy showing TRPV1 receptor expression in the ARC of WT and TRPV1 KO mice. Scale bar: 100 μm. **(C)** TRPV1 receptor expression in the ARC of WT and TRPV1 KO mice. **(D)** Images of fluorescence confocal microscopy showing TRPV1 receptor (red) expression in the ARC of POMC-eGFP mice. Arrowheads represent TRPV1-positive ARC POMC neurons. Scale bar: 20 μm. **(E)** Images of fluorescence confocal microscopy showing TRPV1 receptor-positive POMC neurons (red) in the ARC of TRPV1-Cre mice injected with AAV5-DIO-GFP (Arrowheads). Approximately 90% of GFP-positive cells contained POMC (*n* = 357 out of 401 GFP-positive cells, *n* = 3 mice). Scale bar: 20 μm. **(F)** Representative traces showing depolarization of POMC neurons in response to increased temperature. This depolarization was abolished by the TRPV1 receptor antagonist and by knockdown of TRPV1 receptors with TRPV1 receptor shRNA. Scale bar: 25 mV, 1 min. **(G)** Pooled data of mean membrane potential in response to elevated bath temperature from 34 °C to 38 °C (Control, *n* = 10 neurons, AMG9810 [10 μM], *n* = 11 neurons, and Trpv1 shRNA, *n* = 11 neurons, *p < 0.05, **p < 0.01, ***p < 0.001 versus 34 °C). **(H)** Pooled data of mean membrane potential at 34 °C and 38 °C (38 °C, Control, ΔVm, 11.6 ± 2.1 mV, *n* = 10 neurons, AMG9810, ΔVm, 2.5 ± 0.5 mV, *n* = 11 neurons, TRPV1 shRNA, ΔVm, 3.6 ± 1.0 mV, *n* = 11 neurons, **p < 0.01, ***p < 0.001 versus 34 °C). Data are shown as mean ± SEM. AAV5, adeno-associated virus serotype 5; ARC, arcuate nucleus of the hypothalamus; DIO, double-floxed inversed open reading frame; GFP, green fluorescent protein; KO, knock-out; POMC, proopiomelanocortin; RT-PCR, reverse transcription-polymerase chain reaction; shRNA, short hairpin RNA; TRPV1, transient receptor potential vanilloid 1 receptor; VMH, ventromedial nucleus of the hypothalamus; WT, wild-type; 18S, 18S ribosomal RNA.

We next performed single-cell reverse transcription-polymerase chain reaction (RT-PCR) and immunohistochemistry to identify the expression of capsaicin-sensitive TRPV1 receptors in ARC POMC neurons. Single-cell RT-PCR analysis of ARC POMC neurons revealed that approximately two thirds of ARC POMC neurons examined contained *Trpv1* messenger RNA (mRNA) (Fig 1A, *n* = 21 out of 34 neurons). In addition to the expression of *Trpv1* transcripts, some ARC POMC neurons expressed *Trpv3* and *Trpv4* mRNA (S2 Fig). The prior transcriptome study in which GFP-tagged POMC neurons were isolated at room temperature (24 °C) showed the lack of *Trpv1* mRNA in ARC POMC neurons [17]. We further investigated *Trpv1* mRNA expression in ARC POMC neurons from hypothalamic slices kept at room temperature. We found that this low temperature treatment strongly down-regulated *Trpv1* mRNA levels, although the number of ARC POMC neurons expressing glutamate decarboxylase 2 (*Gad2*) mRNA was similar to that observed at 36 °C (S3 Fig). Thus, methodological differences in the temperatures at which the tissue was isolated may account for prior failures to identify *Trpv1* transcripts in ARC POMC neurons.

Immunostaining of ARC POMC neurons with an anti-TRPV1 receptor antibody showed TRPV1 receptor-positive cells in the ARC of control animals (Fig 1B and S4A Fig). However, no labeled cells were observed in TRPV1 knock-out (KO) mice (Fig 1B). Additionally, western blot analysis showed a TRPV1-positive signal in lysates from the ARC of control but not TRPV1 KO mice (Fig 1C). Importantly, immunostaining, quantitative polymerase chain reaction (qPCR), and western blot analysis showed no expression of TRPV1 receptors around the ARC (S4B, S4C and S4D Fig). All these results provide the specificity of the anti-TRPV1 antibodies that we used in this study.

Double immunolabeling experiments with an anti-POMC antibody revealed that most TRPV1 receptor-positive cells in the ARC were POMC-containing neurons (Fig 1D, 96.3 ± 0.3%; *n* = 3 mice), consistent with our neurophysiological results. In contrast, very few neuropeptide Y-expressing neurons in the ARC expressed TRPV1 receptors (S5A Fig; 1.6 ± 0.7% of TRPV1-postive neurons; *n* = 3 mice). To further examine the expression of TRPV1 receptors in ARC POMC neurons, we used the TRPV1-Cre knock-in animal model [12]. An adeno-associated virus serotype 1 (AAV1) encoding Cre-inducible GFP (AAV1-DIO-GFP) was injected into the ARC of TRPV1-Cre mice. Two weeks post viral injections, we immunostained hypothalamic sections with anti-GFP and anti-POMC antibodies. We found that the ARC contained GFP-expressing cells in the ARC, confirming the expressing of TRPV1-positive cells in this area. Importantly, GFP-positive cells also expressed POMC (Fig 1E). These results from the application of molecular, genetic, and immunohistochemical approaches strongly support the interpretation that a population of ARC POMC neurons expresses capsaicin-sensitive TRPV1 receptors and provide our rationale for subsequent investigation of the roles of TRPV1 receptors in ARC POMC neurons in the regulation of feeding in mice.

### ARC POMC neurons express functional temperature-sensitive TRPV1-like receptors

Increased temperature activates capsaicin-sensitive TRPV1 receptors [4]. We examined the neurophysiological impact of temperature on TRPV1-expressing POMC neuronal activity. Whole-cell voltage-clamp recordings were obtained from ARC POMC neurons in the presence of synaptic blockers (CNQX, 10 μM, and picrotoxin, 100 μM) and TTX (0.5 μM). Raising bath temperature from 34 °C up to 38 °C elicited an inward current in ARC POMC neurons (S6A and S6B Fig). In addition, these thermal stimuli significantly depolarized ARC POMC neurons in current-clamp configurations in the presence of synaptic blockers (Fig 1F and 1G and S6C Fig). Approximately 40% of POMC neurons did not respond to increased temperature (S6D and S6E Fig). This depolarization was blocked by the TRPV1 receptor antagonist AMG9810 and by *Trpv1* gene knockdown with short hairpin RNA (shRNA) in the ARC (Fig 1F, 1G and 1H). In contrast, NPY neurons did not respond to increased temperature (S5D and S5E Fig). It has been demonstrated that recombinant TRPV1 receptors responded only to noxious temperatures (>42 °C) [4]. Interestingly, TRPV receptors could coassemble into heteromeric channels, and heteromeric TRPV1 receptors with other TRPV subunits had altered temperature sensitivity [23,29]. For instance, TRPV1/TRPV3 channels had a lower threshold temperature, around 34 °C [23]. Importantly, the sensitivity to capsaicin of heteromeric TRPV channels was determined by the presence of TRPV1 subunits [23]. As ARC POMC neurons had *Trpv3* and *Trpv4* mRNA along with *Trpv1* mRNA, we examined whether knockdown of the *Trpv3* and *Trpv4* genes in the ARC affect thermal sensitivity. POMC neurons in mice injected with *Trpv3* and *Trpv4* shRNA were less sensitive to increased temperature (S7A and S7B Fig). Therefore, our results suggest that ARC POMC neurons express TRPV1 receptor subunit-containing heteromeric TRPV channels (hence, we describe them as “TRPV1-like”).

### Capsaicin-induced depolarization reduces food intake

**Fig 2:**
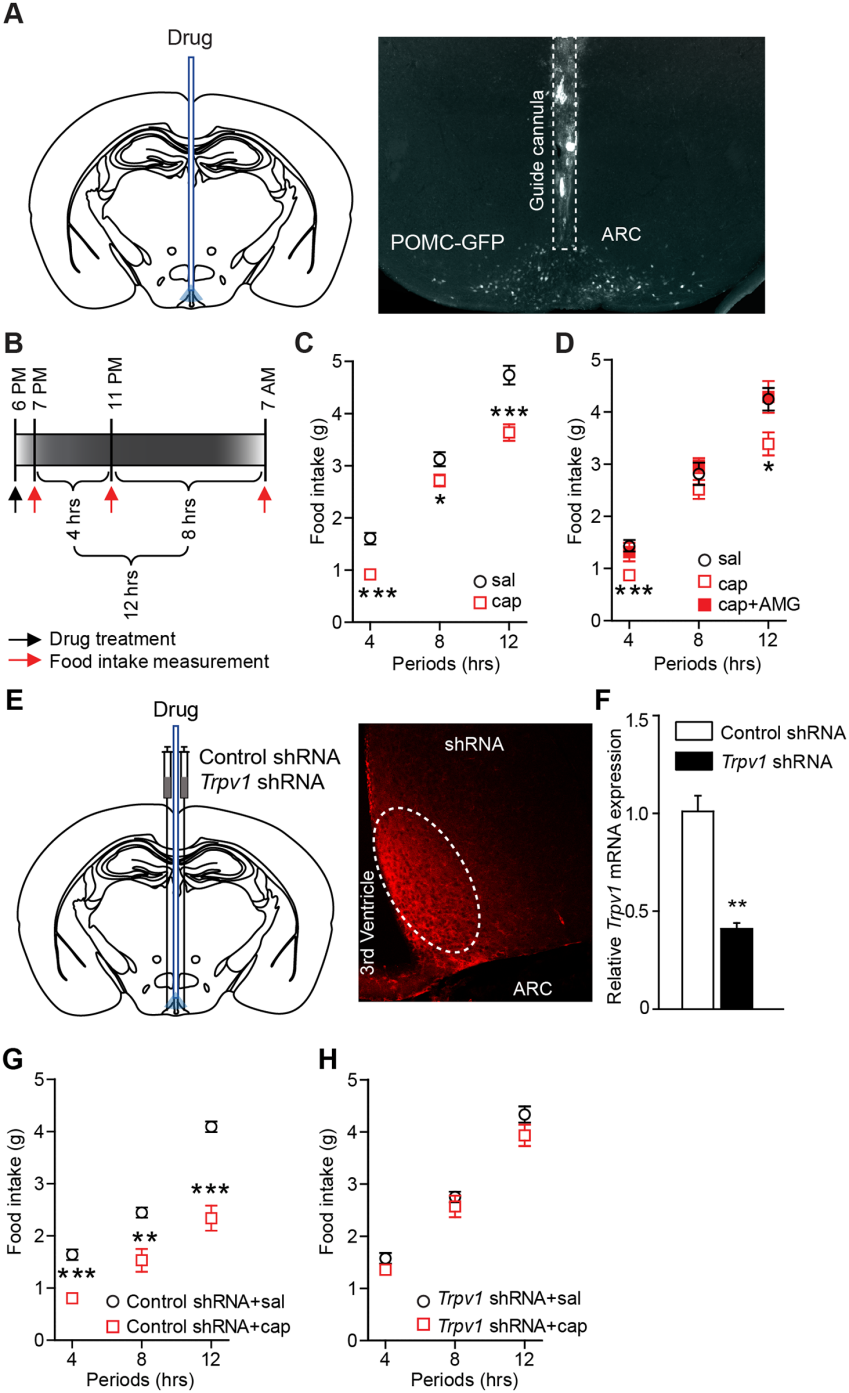
Activation of TRPV1 receptors in the ARC reduces food intake. **(A)** Schematic drawing of our experimental configuration (left panel). Saline and drug were injected into the ARC of POMC-GFP mice (right panel). **(B)** Scheme depicting food intake measurement. **(C)** Pooled data from 17 mice of food intake following local microinjection of capsaicin into the ARC. *p < 0.05, ***p < 0.001. **(D)** Pooled data from 7 mice showing blockade of the effect of capsaicin with AMG9810 (3 μg/μL). ***p < 0.001, *p < 0.05. **(E)** Schematic drawing of our experimental configuration. shRNAs were bilaterally injected into the ARC (left panel). Images of fluorescence microscopy showing transfection of control shRNA in the ARC (right panel). White oval circle shows shRNA injection site. **(F)** *Trpv1* mRNA expression in mice injected with control and *Trpv1* shRNA. mRNAs were extracted from the ARC of mice injected with control (*n* = 6 animals) and *Trpv1* shRNA (*n* = 6 animals). *Tprv1* mRNA expression was significantly reduced by injection of TRPV1 shRNA (**p < 0.01). **(G** and **H)** Pooled data showing that capsaicin no longer reduces food intake in mice injected with *Trpv1* shRNA into the ARC (control sgRNA, *n* = 9 mice; *Trpv1* sgRNA, *n* = 9 mice). **p < 0.01, ***p < 0.001. Data are shown as mean ± SEM. ARC, arcuate nucleus of the hypothalamus; cap, capsaicin; GFP, green fluorescent protein; POMC, proopiomelanocortin; sal, saline; sgRNA, single guide RNA; shRNA, short hairpin RNA; TRPV1, transient receptor potential vanilloid 1 receptor.

Given that ARC POMC neurons play a role in the control of food intake [30–34], we asked whether activation of TRPV1-like receptors in ARC POMC neurons is sufficient to reduce food intake. To test this possibility, animals were fasted for 6 h (from 1 PM to 7 PM) before drug infusion and capsaicin was directly infused into the ARC. Local microinjection of capsaicin (100 nM) significantly reduced food intake (Fig 2A, 2B and 2C). This anorexigenic effect was blocked by local injection of AMG9810 (Fig 2D). Genetic silencing of the *Trpv1* gene by injecting *Trpv1* shRNA into the ARC also abolished the effect of capsaicin (Fig 2E, 2F, 2G, 2H and S1 Table). Therefore, activation of capsaicin-sensitive TRPV1-like receptors in the ARC decreases food intake.

**Fig 3:**
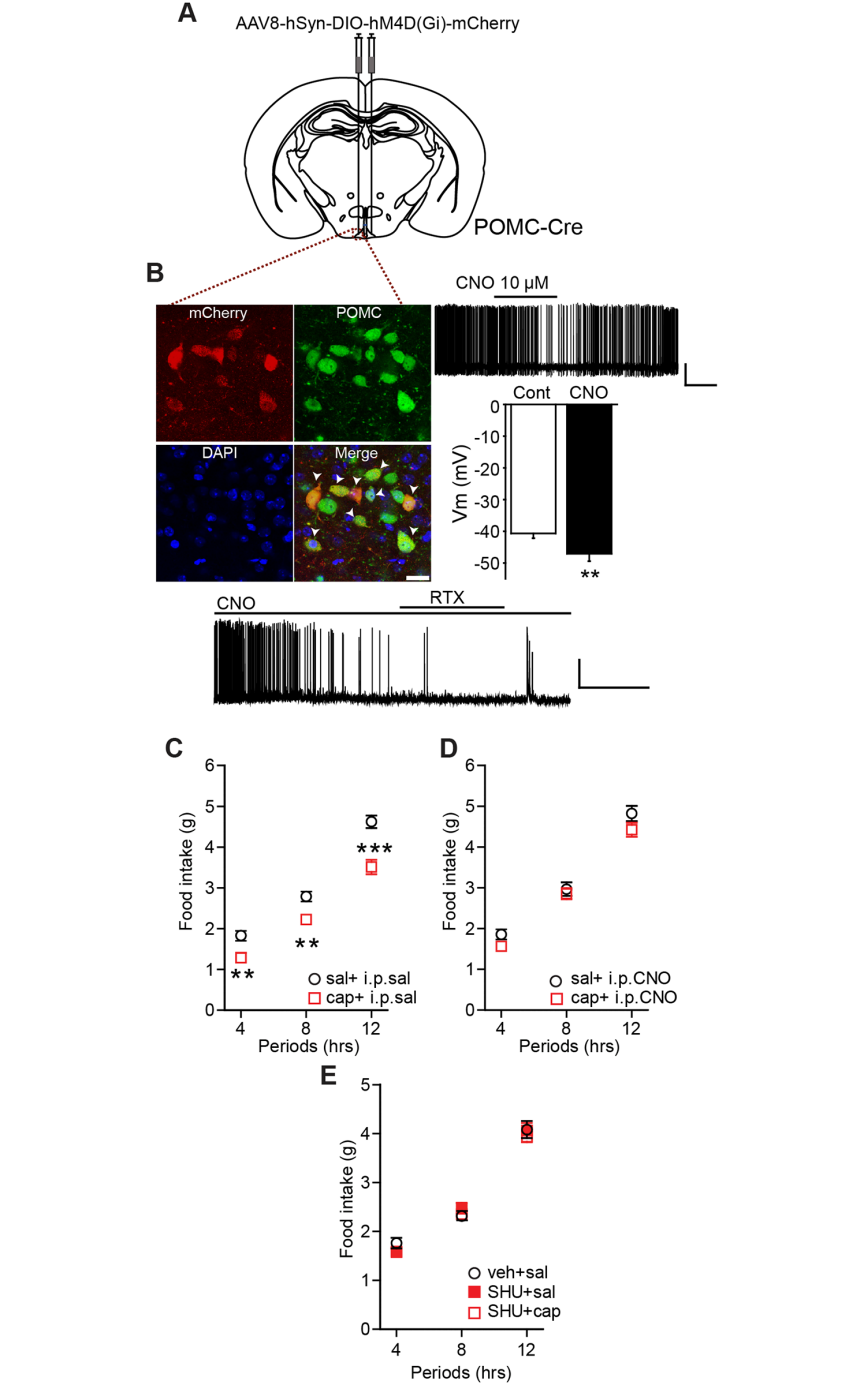
Activation of TRPV1 receptors in ARC POMC neurons reduces food intake. **(A)** Schematic drawing of our experimental configuration showing that Cre-inducible AAV-G_i/o_-DREADD was injected into the ARC of POMC-Cre mice. **(B)** Expression of G_i/o_-DREADD in POMC neurons in the ARC (top left panel, scale bar: 20 μm). Arrowheads represent POMC neurons that co-expressed mCherry (*n* = 493 out of 529 mCherry-positive neurons, *n* = 3 mice). Treatment with CNO (10 μM) hyperpolarized POMC neurons (top right panel; Control, −40.7 ± 1.5 mV; CNO, −47.1 ± 2.3 mV, *n* = 6 neurons, **p < 0.01). Scale bar: 25 mV, 2 min. Bottom panel: In the presence of CNO, treatment of the TRPV1 agonist was not able to depolarize POMC neurons (*n* = 3 neurons). Scale bar: 20 mV, 100 s. (**C** and **D**) Pooled data from 8 mice showing blockade of the effect of capsaicin by activation of G_i/o_-DREADD in ARC POMC neurons. **p < 0.01, ***p < 0.001. **(E)** Pooled data from 11 mice showing blockade of the effect of capsaicin by the MC3/4 receptor antagonist SHU9119 (5 μM). *p* > 0.05. Data are shown as mean ± SEM. AAV, adeno-associated virus; ARC, arcuate nucleus of the hypothalamus; cap, capsaicin; CNO, clozapine-N-oxide; Cre, Cre recombinase; DREADD, designer receptor exclusively activated by designer drugs; hSyn, human synapsin I promoter; i.p., Intraperitoneal; mCherry, monomeric cherry fluorescent protein; MC3/4, melanocortin 3/4; POMC, proopiomelanocortin; RTX, resiniferatoxin; sal, saline; TRPV1, transient receptor potential vanilloid 1 receptor.

We then examined whether capsaicin-induced depolarization of ARC POMC neurons reduces food intake. We expressed inhibitory designer receptors exclusively activated by designer drugs (DREADDs) in ARC POMC neurons by injection of a Cre-dependent AAV5-G_i/o_-DREADD- monomeric cherry fluorescent protein (mCherry) into the ARC of the POMC-Cre animals (Fig 3A). Two weeks after such virus injections, there was expression of mCherry-positive cells in the ARC (Fig 3B). More than 90% of mCherry positive cells contained POMC (Fig 3B). Treatment with clozapine-N-oxide (CNO) (10 μM) inhibited the activity of mCherry-positive POMC neurons and blocked the effect of RTX in hypothalamic slice preparations (Fig 3B). In in vivo studies of these animals, intraperitoneal administration of CNO alone did not change food intake as described in prior studies [35]. However, CNO-induced inhibition of ARC POMC neurons was sufficient to block the effect of capsaicin on food intake (Fig 3C and 3D and S1 Table), suggesting that inhibition of ARC POMC neurons counteracts capsaicin-induced excitation of ARC POMC neurons.

ARC POMC neurons synthesize and release POMC-derived peptides such as anorexigenic α-melanocyte-stimulating hormone (α-MSH) [36,37] and depolarization of ARC POMC neurons releases α-MSH, resulting in reduced food intake [36]. We examined whether the anorexigenic effect of capsaicin is due to the release of α-MSH. We intracerebroventricularly injected the melanocortin 3/4 receptor antagonist SHU9119 prior to local injection of capsaicin. While injection of SHU9119 had no feeding effect when administered alone (Fig 3E), pretreatment with SHU9119 abolished the anorexigenic effect of capsaicin (Fig 3E and S1 Table). In contrast, capsaicin was still effective in reducing feeding in the presence of the antagonist for the opioid receptor naloxone (1 mM; S8 Fig). Our results support the interpretation that activation of TRPV1-like receptor induces the release of anorexigenic α-MSH from ARC POMC neurons and consequently reduces food intake.

### Activation of TRPV1-like receptors expressed in POMC neurons reduces feeding

**Fig 4:**
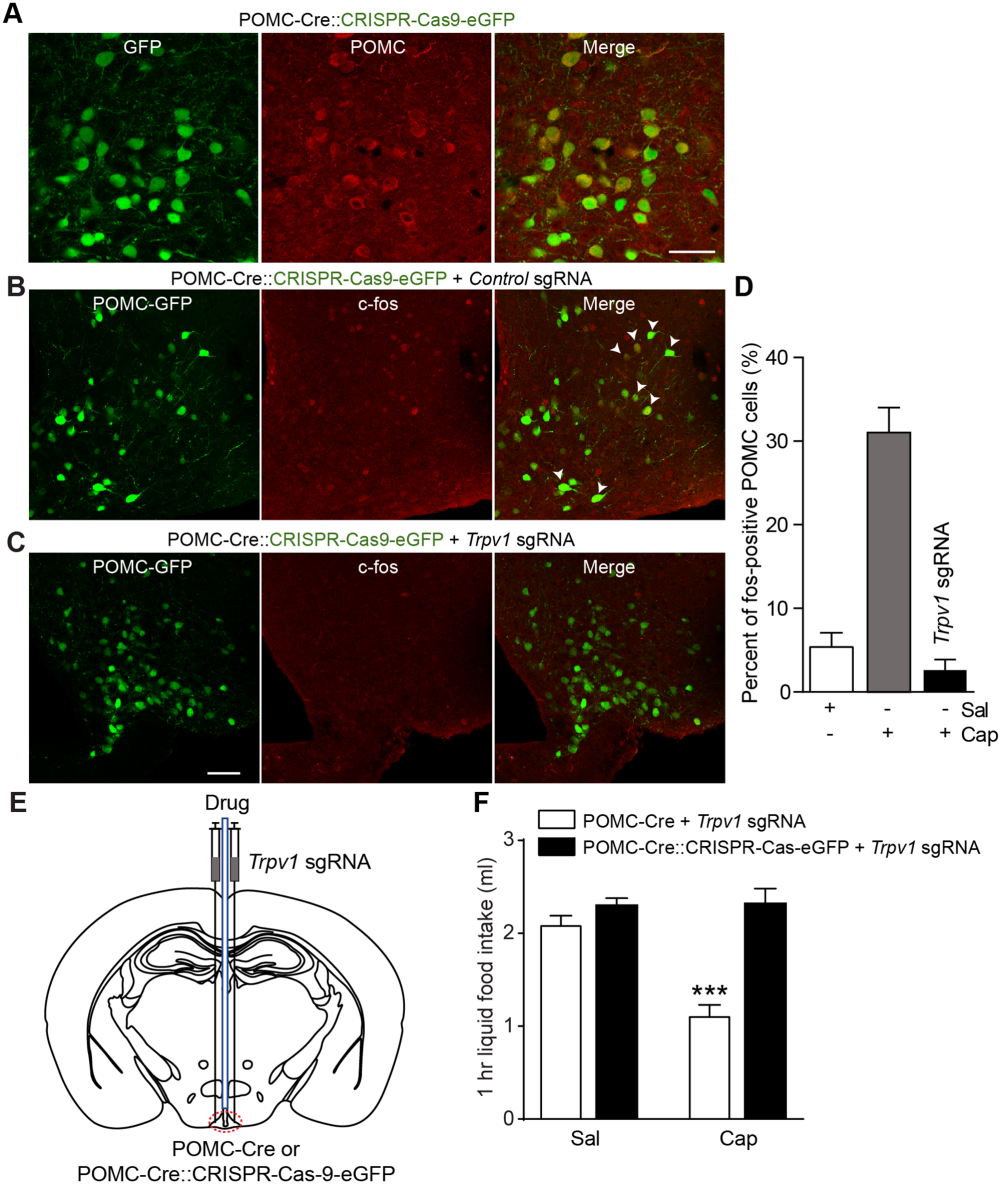
Knockdown of the *Trpv1* gene in ARC POMC neurons blocks the anorexigenic effect of capsaicin. **(A)** Images of fluorescence confocal microscopy showing GFP-positive POMC neurons in the ARC of POMC-Cre::CRISPR-Cas-9-eGFP mice. Scale bar: 50 μm. **(B)** Images of fluorescence confocal microscopy showing that local injection of capsaicin induced expression of c-fos protein in POMC neurons in the ARC. **(C)** Images of fluorescence confocal microscopy showing that local injection of capsaicin did not induce c-fos protein expression in mice lacking TRPV1 receptors in ARC POMC neurons. Scale bar: 50 μm. **(D)** Pooled data show the percentage of POMC neurons expressing c-fos protein in control mice and mice lacking TRPV1 receptors in ARC POMC neurons following treatment with saline and capsaicin (*n* = 5, 5, and 3 animals, respectively). **(E** and **F)** Schematic drawing of our experimental configuration. *Trpv1* sgRNA was bilaterally injected into the ARC of POMC-Cre and POMC-Cre::CRISPR-Cas-9-eGFP mice (**E**). Direct injection of capsaicin into ARC did not reduce food intake in mice silencing the *Trpv1* gene in ARC POMC neurons (**F**) (*n* = 6 and 7 mice, respectively). Mice were fasted for 6 h (1 PM to 7 PM) ***p < 0.001. Data are shown as mean ± SEM. ARC, arcuate nucleus of the hypothalamus; cap, capsaicin; Cre, Cre recombinase; CRISPR-Cas-9, clustered regularly interspaced short palindromic repeats/CRISPR-associated protein 9; eGFP, enhanced green fluorescent protein; GFP, green fluorescent protein; POMC, proopiomelanocortin; sal, saline; sgRNA, single guide RNA; TRPV1, transient receptor potential vanilloid 1 receptor.

Next, we investigated whether activation of TRPV1-like receptors expressed exclusively in ARC POMC neurons regulates feeding. To this end, we used the Cre-dependent clustered regularly interspaced short palindromic repeats/CRISPR-associated protein 9 (CRISPR/Cas-9) system to silence the *Trpv1* gene in ARC POMC neurons. We crossed POMC-Cre with floxed-stop CRISPR/Cas-9-eGFP mice to generate POMC-Cre;;CRISPR/Cas-9-eGFP mice (Fig 4A). We injected *Trpv1* single guide RNA (sgRNA) into the ARC of these mice and then examined whether ARC POMC neurons still respond to capsaicin. Immunohistochemical experiments with an anti-c-fos protein antibody revealed that local injection of capsaicin induced c-fos protein expression in ARC POMC neurons in control mice, whereas mice injected with *Trpv1* sgRNA to ARC POMC neurons showed no c-fos protein expression in response to capsaicin (Fig 4B, 4C and 4D). Moreover, the mean amplitude of temperature-sensitive currents in POMC neurons from mice injected with *Trpv1* sgRNA was significantly smaller than that observed in Controls (S6A and S6B Fig).

**Fig 5:**
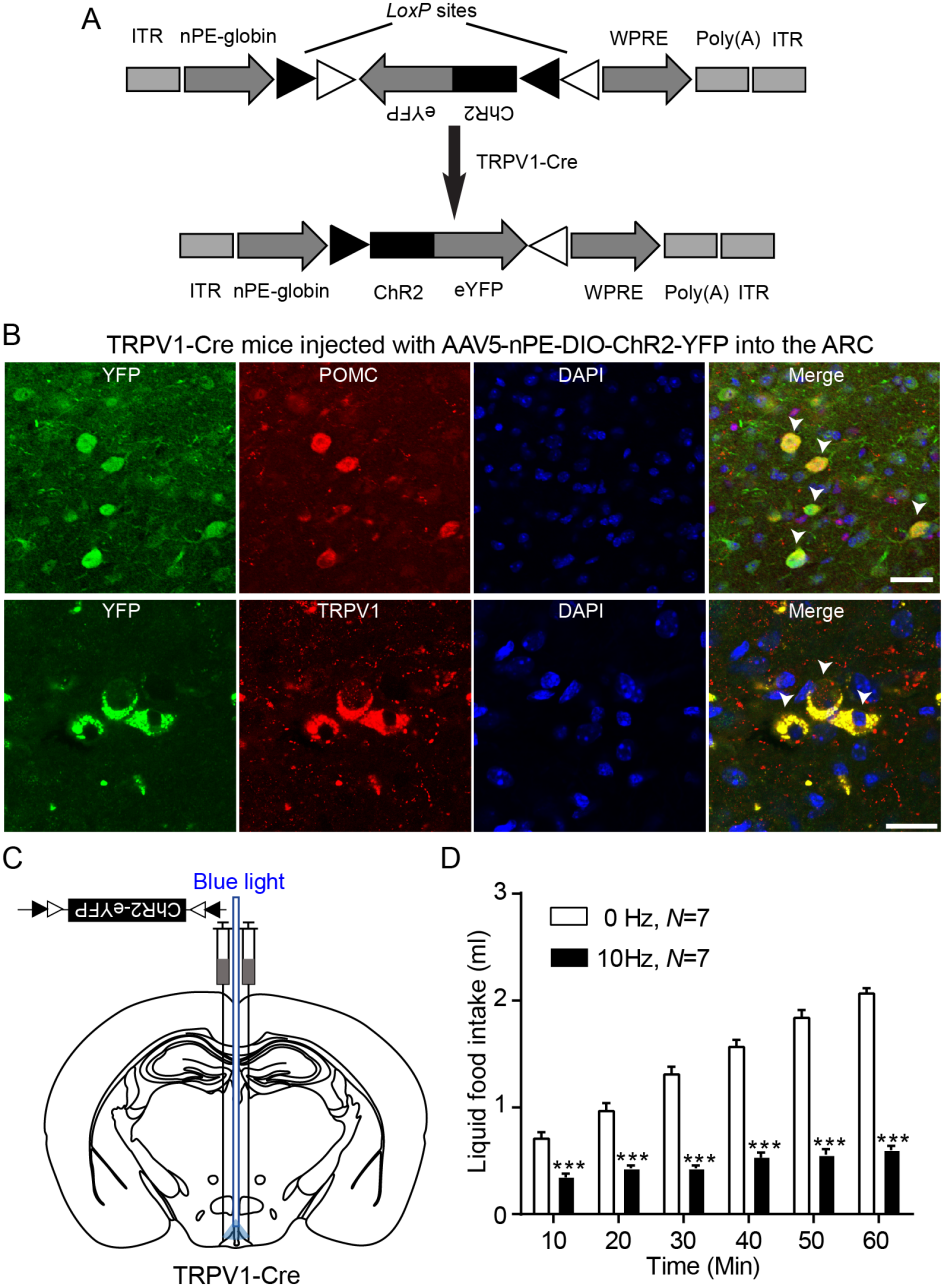
Activation of TRPV1-like receptor-expressing POMC neurons reduces feeding. **(A)** Schematic drawing of the double-floxed Cre-dependent AAV vector expressing ChR2–eYFP under the control of the nPE-globin. **(B)** Images of confocal microscopy showing co-expression of POMC and YFP in the ARC of TRPV1 Cre mice injected with AAV5-nPE-globin-DIO-ChR2-eYFP in the ARC (arrowheads; top panel). Bottom panel: Co-expression of TRPV1 and YFP (arrowheads). Scale bar: 20 μm. **(C)** Schematic drawing of our experimental configuration. AAV5-nPE-ChR2 viruses were bilaterally injected into the ARC of TRPV1-Cre mice. An optic cannula was implanted to the ARC to optogenetically stimulate TRPV1-like receptor-expressing POMC neurons. **(D)** Pooled data showing effect of optogenetic stimulation of TRPV1-like receptor-expressing POMC in the ARC. Ten-hertz stimulation significantly reduced food intake for 1 h (without stimulation, 2.1 ± 0.04 mL; with stimulation, 0.6 ± 0.04 mL, *n* = 7 mice, ***p < 0.001). Data are shown as mean ± SEM. AAV, adeno-associated virus; ChR2, channelrhodopsin-2; Cre, Cre recombinase; DIO, double-floxed inversed open reading frame; eYFP, enhanced YFP; ITR, inverted terminal repeat; *LoxP*, locus of X(cross)-over in P1; nPE, neuronal POMC enhancer; POMC, proopiomelanocortin; TRPV1, transient receptor potential vanilloid 1 receptor; TTX, tetrodotoxin; WPRE, woodchuck hepatitis virus posttranscriptional regulatory element; YFP, yellow fluorescent protein.

Given that the anorexigenic effect of capsaicin was abolished by the melanocortin 3 and 4 receptor (MC3/4R) antagonist, we expected that administration of capsaicin would not reduce food intake in mice silencing the *Trpv1* gene in ARC POMC neurons. Indeed, capsaicin had no effect on feeding in this animal model, whereas POMC-Cre mice injected with *Trpv1* sgRNA still showed a reduction in food intake following capsaicin treatment (Fig 4E and 4F). Our results appeared to be inconsistent with prior studies showing that stimulation of the whole population of ARC POMC neurons failed to decrease food intake [35,36,38]. We thus investigated whether activating TRPV1-like receptor-expressing ARC POMC neurons decreases food intake. To selectively stimulate TRPV1-like receptor-expressing POMC neurons in the ARC, we generated an AAV5 carrying a Cre-dependent channelrhodopsin-2 (ChR2)–enhanced yellow fluorescent protein (eYFP) expression cassette under the control of the two neuronal POMC enhancers (nPE1 and nPE2) (AAV5-nPE-DIO-ChR2-YFP, Fig 5A). These enhancers have been shown to drive reporter gene expression to ARC POMC neurons [39]. We injected this viral vector into the ARC of TRPV1-Cre mice with an expectation that only TRPV1-like receptor-expressing POMC neurons, among the entire ARC POMC neuronal population, would express ChR2. Eight weeks post viral injections, we found expression of the reporter protein in POMC neurons in the ARC (Fig 5B). Importantly, optogenetic stimulation of these neurons at 10 Hz for 1 h significantly reduced food intake (Fig 5C and 5D). Our results support the interpretation that, contrary to the prior findings [35,36,38], selective stimulation of a specific subset of ARC POMC neurons (i.e., TRPV1-like receptor-expressing) is sufficient to decrease food intake.

### A rise in body temperature elevates ARC temperature

**Fig 6:**
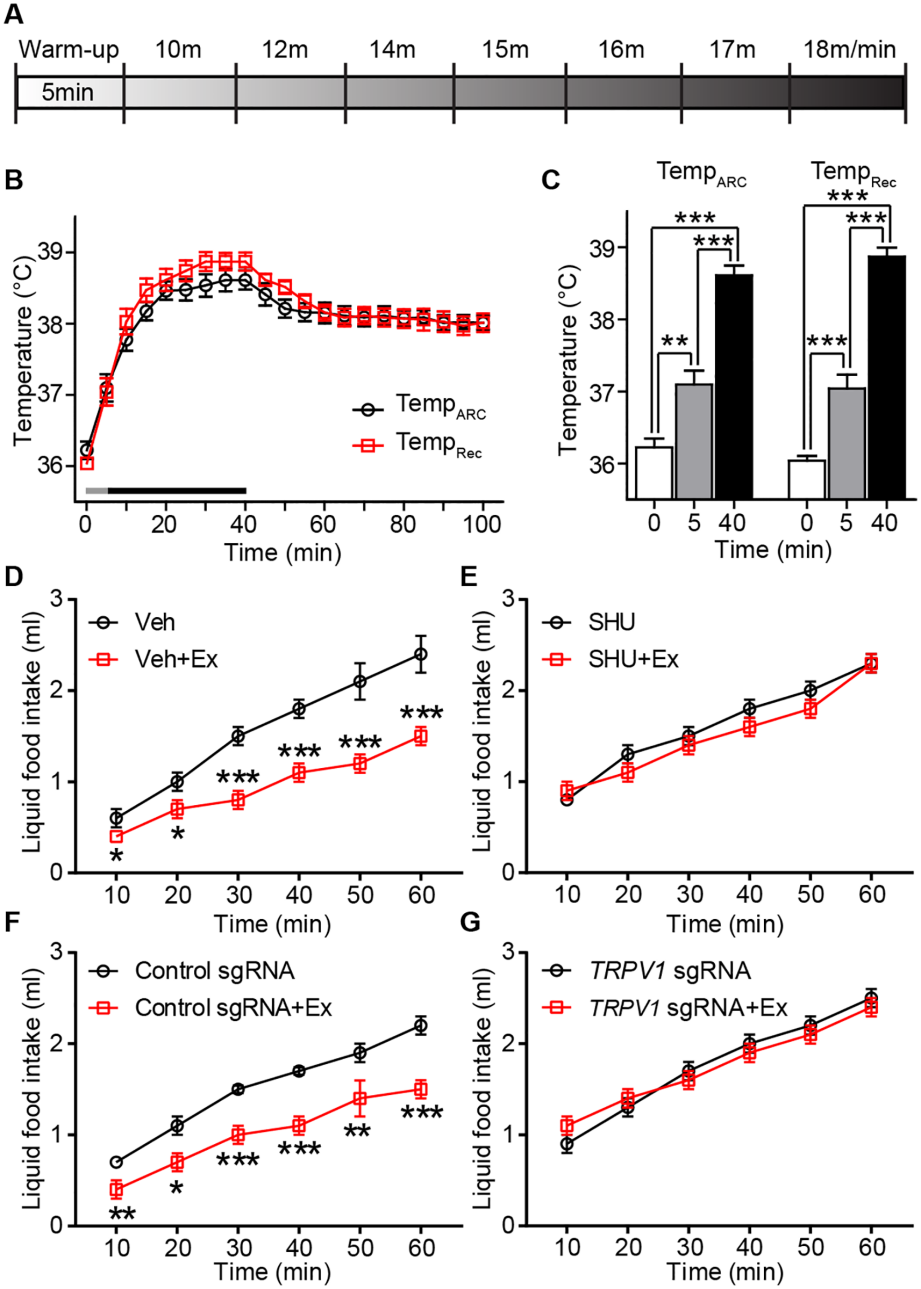
Exercise increases body and ARC temperatures. **(A)** Scheme depicting the mouse treadmill protocol. Mice were always warmed up for 5 min at 7 m/min and the speed was slowly ramped up to 18 m/min. **(B** and **C)** Pooled data showing changes in body and ARC temperature during treadmill running (gray line, warming up for 5 min and black line, running for 35 min [**B**]). Plot showing mean temperature at 0, 5, and 40 min exercise (at 40 min, mean body temperature, 38.9 ± 0.1 °C; mean ARC temperature, 38.6 ± 0.2 °C). **(D)** Pooled data from 9 mice showing the effect of exercise on liquid food intake for 1 h (*p < 0.05, ***p < 0.001). **(E)** Pooled data from 9 mice showing the effect of exercise on food intake for 1 h with an MC3/4R antagonist. Exercise-induced anorexigenic effect was abolished by local injection of SHU9001. **(F)** Pooled data from 9 mice showing the effect of exercise on food intake for 1 h in mice injected with control sgRNA. Exercise-induced anorexigenic effect is absent in mice silencing the *Trpv1* gene in ARC POMC neurons. *p < 0.05, **p < 0.01, ***p < 0.001. **(G)** Pooled data from 9 mice showing the effect of exercise on food intake for 1 h in mice silencing the *Trpv1* gene in POMC neurons. Exercise no longer reduced food intake in mice lacking the *Trpv1* gene in POMC neurons. Data are shown as mean ± SEM. ARC, arcuate nucleus of the hypothalamus; Ex, exercise; MC3/4R, melanocortin 3 and 4 receptor; POMC, proopiomelanocortin; sgRNA, single guide RNA; *Trpv1*, transient receptor potential vanilloid 1 receptor; Veh, vehicle.

A small elevation in temperature within the physiological range (from 37 °C to 38 °C) activated TRPV1-like receptors in ARC POMC neurons in hypothalamic slices. We asked whether ARC temperature undergoes such elevations under normal physiological conditions. It has been shown that physical exercise raises not only body but also brain temperature [40]. Thus, we measured both ARC and body temperature during a 40-min bout of treadmill running in trained mice (Fig 6A and 6B and S1 Movie). This exercise rapidly raised body as well as ARC temperature (Fig 6B and 6C). ARC temperature reached a plateau after 20 min exercise and remained high for more than 1 h.

We sought to determine if this rise in ARC temperature is associated with reduced feeding through activation of TRPV1-like receptors in ARC POMC neurons. Mice were given liquid food following the completion of the exercise bout to measure acute changes in food intake (Fig 6D). We found that exercised mice consumed significantly less food than did non-exercised mice (Fig 6D). Importantly, the anorexigenic effect of exercise was abolished by administration of the MC3/4R antagonist SHU9119 (Fig 6E and S2 Table), suggesting that exercise-induced rise in ARC temperature activates ARC POMC neurons. To further determine this possibility, we measured food intake in mice silencing the *Trpv1* gene in ARC POMC neurons after exercise. We found that, although mice injected with control sgRNA showed a decrease in food intake (Fig 6F), exercise had no effect on food intake in mice silencing the *Trpv1* gene in ARC POMC neurons (Fig 6G and S2 Table). Hence, our results propose that elevated hypothalamic temperature can activate temperature-sensitive TRPV1-like receptors in ARC POMC neurons, which may result in a reduction in food intake.

## Discussion

All the results from neurophysiologic, molecular, genetic, and immunohistochemical studies provided evidence that ARC POMC neurons expressed functional TRPV1 channels. ARC POMC neurons were sensitive to capsaicin but activated by physiological warm temperature around 37 °C, suggesting that ARC POMC neurons may express heteromeric rather than homomeric TRPV1 receptors. In fact, a subset of ARC POMC neurons had *Trpv3* and *Trpv4* transcripts. Pharmacological and optogenetic stimulation of TRPV1-like receptor-expressing ARC POMC neurons reduced food intake. This anorexigenic effect was blocked by an MC3/4R antagonist. Importantly, a transient increase in body temperature was rapidly transmitted to the ARC. This elevation of hypothalamic temperature was enough to reduce food intake, although temperature-independent effects might explain these anorexigenic effects. Mice silencing the *Trpv1* gene exclusively in ARC POMC neurons showed no effect of increased hypothalamic temperature on food intake. Therefore, our results support the interpretation that ARC POMC neurons have the ability to respond not only to hormones and nutrients but also to changes in body temperature.

At first glance, our present TRPV1 receptor expression results may appear to be inconsistent with those from a prior study describing the lack of *Trpv1* mRNA expression in ARC POMC neurons [17]. We think this is likely due to methodological differences in the temperatures applied during transcriptome assessments. In the study of cell type–specific transcriptomics, POMC-GFP cells were sorted at room temperature [17]. Importantly, such subphysiological temperatures reduced*Trpv1* mRNA expression [41]. Thus, transcriptomic expression of TRPV1 receptors would be expected to be greatly down-regulated under such low temperature conditions; indeed, in our hands we found that low temperature treatment (i.e., 24 °C) strongly decreased *Trpv1* mRNA expression in ARC POMC neurons. We did find robust *Trpv1* mRNA in ARC POMC neurons, but only when we incubated at a temperature in the physiological range.

The use of multiple independent techniques, including patch-clamp recording, single-cell RT-PCR, immunohistochemistry, western blotting, gene knockdown with shRNA, CRISPR/Cas-9-mediated gene knockdown, TRPV1 KOs, and TRPV1-Cre mice, provided converging lines of evidence for the expression of TRPV1 receptor subunits in ARC POMC neurons. However, it appears that there was a discrepancy between our results and the prior findings described in the literature [24,25]. In particular, ARC POMC neurons were depolarized by warm temperature (>37 °C). It is unlikely that this depolarization was mediated via activation of homomeric TRPV1 receptors. In fact, recombinant TRPV1 receptors were activated at much higher temperatures (>42 °C) [4,23]. These findings suggest that warm temperature-elicited depolarization may be due to the activation of other than TRPV1 channels, such as TRPV3 and TRPV4 subunits, with a low threshold temperature [5,6,25,42,43]. Although we cannot rule out this possibility, the sensitivity to capsaicin suggests that ARC POMC neurons contained TRPV1 subunits that have a specific binding site for capsaicin [20–22].

It has been documented that TRPV channels can assemble into heteromeric complexes [23,29,44]. Heteromeric TRPV channels containing TRPV1 subunits are always sensitive to capsaicin, but have altered threshold temperature [23,44]. For instance, heteromeric TRPV1/TRPV3 channels are activated at lower temperatures compared to homomeric TRPV1 channels in human embryonic kidney 293 cells (HEK293) cells (>34 °C versus >38 °C) [23], suggesting that ARC POMC neurons may express heteromeric TRPV channels. In our preparations, *Trpv3* and *Trpv4* mRNA were also detected along with *Trpv1* transcripts in ARC POMC neurons. Interestingly, the neurons in the supraoptic and paraventricular nucleus expressing TRPV1 and TRPV4 receptor subunits were excited by warm temperature [13,16,45]. The neurons in these areas in TRPV1 KO mice still responded to elevated temperature, although the amplitude of temperature-elicited currents was greatly reduced [13,16]. In line with these findings, we found that knockdown of the *Trpv3* and *Trpv4* genes in the ARC altered the thermal sensitivity. Hence, it seems likely that TRPV receptors in the hypothalamus generally form heteromeric rather than homomeric complexes that have capsaicin-sensitive TRPV1 subunits (TRPV1-like).

Despite the well-known anorexigenic effects of α-MSH [46], stimulation of the entire ARC POMC population did not produce the canonical reduction in food intake [35,36,38]. The lack of effects of selective stimulation of ARC POMC neurons would be due in part to POMC neuron heterogeneity. A large and growing body of literature supports the idea that ARC POMC neurons are neurochemically, anatomically, and functionally heterogeneous [19,31,47–51]. For example, activation of ARC POMC neurons by the cannabinoid receptor type 1 agonist has been shown to promote food intake through the release of opioids [31]. In our preparations, optogenetic stimulation of TRPV1-expressing POMC neurons clearly reduced food intake. These findings suggest that neurochemically and/or neuroanatomically distinct subpopulations of ARC POMC neurons play distinct roles in feeding behavior. In fact, while both TRPV1 and cannabinoid type 1 receptor (CB1) agonists depolarize POMC neurons [31], the physiological consequences of ARC POMC neuron activation by TRPV1 versus CB1 agonists diverged in opposite directions. Hence, it is plausible that the net balance between anorexigenic melanocortin POMC and orexigenic opioid POMC neurons would be finely tuned by differential responses to nutrient-related molecules and signaling, as described in our prior study [19]. Interestingly, long- but not short-term stimulation of ARC POMC neurons effectively decreased food intake and this anorexigenic effect was completely absent in A^y^ mice [36], suggesting that long-term stimulation of the entire population of ARC POMC neurons could indeed shift the net balance toward the anorexigenic melanocortin outflow.

Administration of capsaicin reduced food intake and prevented body weight gain in rodents and humans [52,53]. TRPV1 KO mice gained more body weight than did control animals in adulthood and when fed a high-fat diet [54]. Our present findings provided possible explanations as to how vanilloids contribute to the regulation of feeding behavior. In other words, capsaicin and its analog excite TRPV1-like receptor-expressing POMC neurons, resulting in lowered food intake. Importantly, TRPV1-like receptors in ARC POMC neurons were activated not only by vanilloids but also by small, physiological elevations in temperature that can also easily be induced by a brief bout of treadmill exercise. Hence, TRPV1-like receptor-expressing POMC neurons could function as a transducer of thermal stimuli. Like hormones and nutrients, temperature-induced depolarization of ARC POMC neurons drove anorexigenic, melanocortinergic neuronal outflow in our preparations. In the early 1960s, feeding-related activity has been shown to result in an “immediate” increase in brain temperature (within less than 1 min) [55], suggesting that diet, like physical exercise, is able to increase hypothalamic temperature. As capsaicin increases satiety and fullness in humans as well [56], it is plausible that elevated hypothalamic temperature could promote satiety and thereby reduce food intake through activation of TRPV1-like receptors in ARC POMC neurons.

In summary, we identify a melanocortinergic circuit beginning in ARC POMC neurons that links acute elevations in peripheral and brain temperature with acute reductions in food intake via activation of TRPV1-like receptors. Such temperature-sensing mechanisms are ideally situated to mediate the regulation of energy intake and expenditure.

## Materials and methods

### Ethics statement

All mouse care and experimental procedures were approved by the Institutional Animal Care Research Advisory Committee of the Albert Einstein College of Medicine (Protocol number: 20150203) and were performed in accordance with the guidelines described in the NIH guide for the care and use of laboratory animals. Stereotaxic surgery and viral injections were performed under isoflurane anesthesia.

### Animals

All mouse care and experimental procedures were approved by the Institutional Animal Care Research Advisory Committee of the Albert Einstein College of Medicine. Mice used in these experiments included POMC-Cre (stock # 005965), POMC-eGFP (stock # 009593), TRPV1-Cre (stock # 017769), floxed-stop CRISPR/Cas-9-eGFP mice (stock # 024857), and TRPV1-KO (stock # 003770, the Jackson Laboratory). Both male and female mice of mixed C57BL/6, FVB, and 129 strain backgrounds were used.

### Slice preparation and electrophysiological recordings

Transverse brain slices were prepared as described in our previous studies [19]. Slices were equilibrated with an oxygenated artificial cerebrospinal fluid (aCSF) for >1 h at 32 °C before transfer to the recording chamber. The slices were continuously superfused with aCSF at a rate of 1.5 mL/min containing the following (in mM): 113 NaCl, 3 KCl, 1 NaH_2_PO_4_, 26 NaHCO_3_, 2.5 CaCl_2_, 1 MgCl_2_, and 5 glucose in 95% O_2_/5% CO_2_.

All neurophysiological recordings were obtained from bright fluorescent green neurons present in POMC-eGFP mice, except as noted, such as POMC-eGFP neurons in POMC-Cre;;CRISPR/Cas9 mice. The internal solution contained the following (in mM): 130 K-gluconate, 5 CaCl_2_, 10 EGTA, 10 HEPES, 2 MgATP, 0.5 Na_2_GTP, and 10 phosphocreatine. Membrane potentials were recorded in the presence of ionotropic glutamate and GABA_A_ receptor antagonists such as CNQX and picrotoxin (10 μM and 100 μM, respectively) with a Multiclamp 700B. Membrane currents were recorded in the presence of TTX (500 nM) and synaptic blockers. Electrophysiological signals were low-pass filtered at 2–5 kHz, stored on a PC, and analyzed offline with pClamp 10 software (Molecular devices). For each recording, the membrane potentials measured from every 30 s were taken as 1 data point. A total of 10 data points before and after the application of drugs were compared using the unpaired *t* test.

### Stereotaxic surgery, drug infusion, and measurement of food intake

Mice were maintained under isoflurane anesthesia and placed in a stereotaxic apparatus. Under aseptic conditions, sterile custom guide cannulas were stereotaxically implanted into the ARC (AP, −1.2 mm; ML, 0; DV, −5.5 mm) for local injection of drugs (1 μL of 100 nM capsaicin, Sigma-Aldrich, cat # M2028 and AMG9810, Tocris bioscience, cat # 2316) and into the lateral ventricle (AP, +0.85 mm; ML, +0.5 mm; DV, −3.0 mm) for injection of SHU9119 (Tocris bioscience, cat # 3420) or naloxone (Tocris bioscience, cat # 0599). Animals were allowed at least a week to recover from surgery. To measure food intake, animals were separated individually in single cages for 5 d and fasted for 6 h (from 1 PM to 7 PM) before drug infusion. SHU9119 (1 μL of 5 μM) or naloxone (1 μL of 1 mM) was intracerebroventricularly administrated 30 min prior to microinjection of capsaicin or saline. Capsaicin and/or AMG9810 were directly infused into the ARC at 6 PM. Food intake was measured from 7 PM to 11 PM and from 11 PM to 7 AM. The mice used for all of the experiments were randomly assigned to a treatment group. For animal studies, sample sizes were estimated according to our previous experience [19]. Single housed mice with a 20% weight loss were excluded from experiments and analyses. For optogenetic experiments, a mono fiber-optic cannula of 230 μm diameter (Doric Lenses, Inc.) was implanted into the ARC and coupled to a 473-nm DPSS laser (Laserglow Technologies).

### Measurement of body and ARC temperatures, and treadmill exercise

To directly measure both body and hypothalamic temperatures, while mice were running on a treadmill, a wire thermoprobe (IT-24P, Physitemp Instruments) was placed into the ARC (AP, −1.2 mm; ML, 0; DV, −5.5 mm) through a guide cannula and another wire thermoprobe (RET-4, Physitemp Instruments) was inserted into the rectum.

For treadmill exercise (Eco-6M, Columbus Instruments), mice were trained to run on the treadmill for 2 wk. The mice were always warmed up for 5 min at 7 m/min and the speed was slowly ramped up to 18 m/min. To measure food intake following exercise, animals were fasted from 1 PM to 7 PM and given liquid food (Ensure original nutrition shake, Abbott). If at any point during the experiment a mouse became exhausted, the shock grid for that lane was turned off and the mouse allowed to rest.

### shRNA, sgRNA, and G_i/o_-DREADD viral injection

Cre-inducible AAV8-hSyn-DIO-hM4D(Gi)-mCherry was purchased from the UNC vector core (200 nl of 3 × 10^12^ pfu/mL per side). Control, *Trpv1*, *Trpv3*, and *Trpv4* shRNA lentiviruses were purchased from Sigma-Aldrich (cat # SHC012V, 350 nl of 1.8 × 10^7^ TU/mL per side, cat # SHCLNV-NM_001001445, 500 nl of 1.2 × 10^7^ TU/mL per side, cat # SHCLNV-NM_145099, 500 nl of 2.6 × 10^7^ TU/mL per side and cat # SHCLNV-NM_022017, 1.9 × 10^7^ TU/mL per side, respectively). Control and *Trpv1* sgRNA lentiviruses were purchased from ABM (cat # K019 and cat # K4625312, 500 nl of 1 × 10^7^ IU/mL per side). Cre-dependent AAV1-DIO-GFP was purchased from the Penn vector core (200 nl of 1 × 10^13^ pfu/mL per side) and AAV5-nPE-DIO-ChR2-YFP (200 nl of 3 × 10^13^ pfu/mL per side) was packaged by Vigene Biosciences, Inc. (Rockville, MD). Viruses were bilaterally injected into the ARC (AP, −1.3 mm; ML, ± 0.1 mm; DV, −6.0 mm) of POMC-Cre, POMC-Cre::POMC-GFP, POMC-Cre;;CRISPR/Cas-9-eGFP mice, and TRPV1-Cre mice. The investigators were blinded to the group allocation during the experiment.

### Single-cell qPCR following single-cell whole-transcriptome amplification of total RNAs from individual ARC POMC neurons

Single-cell whole transcriptome was prepared with REPLI-g whole-transcriptome amplification (WTA) single-cell kit (Qiagen), as previously described [19,47]. The primers used for qPCR were the following: F5′-tctatgatcgcaggagcatc-3′ and R5′-ggtctttgaactcgctgtca-3′ for *Trpv1* (NM_001001445.2), F5′-atttggagtagcgctggcct-3′ and R5′-gctgaagctgccataggaac-3′ for *Trpv3* (NM_145099.2), F5′-ggaccctggcaagagtgaaa-3′ and R5′-aggaccaacgatccctacga-3′ for *Trpv4* (NM_022017.3), F5′-atgccgagattctgctacagt-3′ and F5′-tccagcgagaggtcgagttt-3′ for *Pomc* (NM_001278581.1), and F5′-gtaacccgttgaaccccatt-3′ and R5′-ccatccaatcggtagtagcg-3′ for *18S rRNA* (XR_877120.2).

### Immunofluorescence staining

Mice were anesthetized with isoflurane and transcardially perfused with preperfusion solution (9 g NaCl, 5 g sodium nitrate, 1,000 u heparin in 1 L distilled water). Brains were incubated in 4% paraformaldehyde overnight at cold room and sectioned with a vibratome in 50 μm on the following day. For immunofluorescence staining, sections were incubated in 10 mM sodium citrate solution (pH 9.0) for 30 min at 80 °C in a water bath. Following the antigen retrieval, the sections were blocked in 0.1 M PBS buffer containing 0.1% triton X-100, 5% normal goat serum or normal donkey serum, and 5% bovine serum albumin for 2 h at room temperature and then incubated with anti-TRPV1 (1:1,000, Alomone Labs, cat # ACC-030), anti-c-fos (1:500; Abcam, cat # ab7963), anti-GFP (1:1,000, Abcam, cat # ab13970), anti-mCherry (1:1,000, Novus Biologicals, cat # NBP2-25157), and anti-POMC (1:1,000, Phoenix, cat # H-029-30 [35]) antibodies for 72 h at cold room. And then sections were washed 3 times in PBS and incubated with Alexa 568 anti-rabbit IgG (1:500; Life Technologies, cat # A10042), Alexa 568 anti-mouse IgG (1:500; Life Technologies, cat # A11004), or Alexa 488 anti-chicken IgG (1:500, Abcam, cat # ab150169) for 2 h at room temperature. Tissues were washed, dried, and mounted with VECTASHIELD media containing DAPI. Images were acquired using a Leica SP2 confocal microscope.

### Western blotting

Total lysates were prepared from the ARC of WT, TRPV1-KO mice, and POMC-Cre;;CRISPR/Cas9-eGFP injected with control or *Trpv1* sgRNA using CelLytic mammalian tissue lysis reagent (Sigma-Aldrich, cat # C3228). Total lysates (50 ug) were separated on 12% SDS-PAGE gels and transferred to the PVDF membrane (Bio-Rad, cat # 1620260). The membrane was incubated in 5% skim milk blocking buffer for 2 h at room temperature and then incubated with anti-TRPV1 (1:1,000, Novus Biologicals, NBP1-97417) and anti-β-actin (1:5,000, Sigma, A5316) antibodies diluted in TBST buffer containing 5% BSA for 24 h at 4 °C. After incubation in primary antibodies, the membrane was washed in TBST buffer and probed with anti-rabbit IgG, HRP-linked antibody or anti-mouse IgG, HRP-linked antibody (1:3,000, Cell Signaling, cat # 7074S and 7076) for 2 h at room temperature. All membranes were washed in TBST buffer, incubated with chemiluminescent substrate (Cell Signaling, cat # 7003), and exposed to X-ray film.

### Generation of AAV-nPEs-ChR2

We subcloned the nPE enhancers with the minimal promoter of the human β-globin gene. The pAAV-EF1α-double floxed-hChR2(H134R)-eYFP-WPRE-HGHpA vector was obtained from addgene (Plasmid # 20298). The EF1α promoter was replaced with the nPE-globin gene. The AAV packaging was done by Vigene Biosciences, Inc. (Rockville, MD).

### Statistics

Statistical analyses were performed using an unpaired *t* test or one-way ANOVA with Tukey multiple comparison test (GraphPAD 4.03 and Origin 8.5). Data were considered significantly different when the *p*-value was <0.05. All statistical results are given as mean ± SEM.

## Supporting information

S1 Table Summary of the effects of capsaicin on food intake. (DOCX)

S2 Table Summary of the effects of increased hypothalamic temperature on food intake. (DOCX)

S1 Fig ARC POMC neurons express capsaicin-sensitive TRPV1 receptors. **(A** and **B)** Representative recording sample of the membrane potential before, during, and after treatment with the TRPV1 agonist. Treatment with capsaicin induced a depolarization of POMC neuron. Pooled data from 20 neurons of membrane potential of POMC neurons following application of Cap (100 nM, ΔVm, 3.8 ± 0.5 mV, ***p < 0.001). **(C** and **D)** Representative recording sample showing effect of RTX (C). (D) Pooled data of membrane potential of POMC neurons following application of RTX (ΔVm, 2.5 ± 1.1 mV, *n* = 10 neurons, *p < 0.05). **(E** and **F)** Recording sample showing the effect of the TRPV1 receptor antagonist AMG9810 (10 μM) (E). AMG9810 completely blocked the effect of capsaicin (*n* = 8 neurons, *p* > 0.05) (F). **(G** and **H)** Representative trace showing depolarization of POMC neurons by RTX in the presence of TTX (1 μM). Pooled data of membrane potential of POMC neurons following application of RTX (H) (*n* = 10 neurons, *p* < 0.05). All neurophysiological recordings were obtained from bright fluorescent green neurons present in POMC-eGFP mice in the presence of picrotoxin (100 μM) and CNQX (10 μM). Scale bar: 20 mV, 2 min. Data are shown as mean ± SEM. ARC, arcuate nucleus of the hypothalamus; Cap, capsaicin; CNQX, 6-cyano-7-nitroquinoxaline-2,3-dione; eGFP, enhanced green fluorescent protein; POMC, proopiomelanocortin; RTX, resiniferatoxin; TRPV1, transient receptor potential vanilloid 1 receptor; TTX, tetrodotoxin.
(PDF)

S2 Fig Expression of *Trpv1*, *Trpv3*, and *Trpv4* transcripts in ARC POMC neurons. ARC, arcuate nucleus of the hypothalamus; lane, cell number; M, PCR marker; P, positive control; POMC, proopiomelanocortin; *Trpv1*, *3*, *4*, transient receptor potential vanilloid 1, 3, 4 receptors.
(PDF)

S3 Fig Expression of *Trpv1* mRNA in ARC POMC neurons under different conditions in vitro. Upper panel: simplified structure of the mRNA (numbered boxes show exons of the *Trpv1* gene) encoding *Trpv1*. Arrows show mRNA regions detected by primer sets (outer, blue; inner, red). Gel images show lack of *Trpv1* mRNA in ARC POMC neurons from slices kept at 24 °C. M: PCR marker (*Trpv1*, 248 bp; *Gad2*, 205 bp; *18S rRNA*, 121 bp). P: positive control single-cell samples were collected from hypothalamic slices kept at 24 °C and 36 °C for 3 h. The initial RT reaction was conducted after pressure ejection of the single-cell samples into freshly prepared RT mix A solution (20 U of RNase OUT, 300 ng of random primers, 0.5% NP-40, and RNase free water). Samples were sonicated in a total volume of 10 μL at 4 °C for 5 min and then incubated for 3 min at 65 °C before addition of 10 μL RT mix B (500 μm dNTP, 1 × RT buffer, 5 mm MgCl_2_, 10 mM DTT, and 200 U of Superscript IIl). The tubes were incubated at 25 °C for 5 min, at 42 °C for 1 h, and at 65 °C for 10 min. Three rounds of amplification were done for the detection of *Trpv1* transcripts (two rounds with the outer primer set and one round with the inner primer set) and two rounds of amplification for the analysis of *Gad2* transcripts. The primers used for qPCR were the following: *Trpv1* outer primer, f5′-catgctcattgctctcatgg-3′ and r5′- aaccagggcaaagttcttcc-3′, *Trpv1* inner primer, f5′-catgggcgagactgtcaac-3′ and r5′- ctgggtcctcgttgatgatg-3′; *Gad2* outer primer, f5′-ggcgatggaatcttttctcct-3′, and *Gad2* inner primer f5′-cgcactctggaagacaatga-3′ and r5′-cgaggcgttcgatttcttcaa-3′. ARC, arcuate nucleus of the hypothalamus; dNTP, deoxynucleotide; DTT, dithiothreitol; *Gad2*, glutamate decarboxylase 2; MgCl_2_, magnesium chloride; POMC, proopiomelanocortin; RT, reverse transcription; *Trpv1*, transient receptor potential vanilloid 1 receptor; U, unit.
(PDF)

S4 Fig Expression of TRPV1 receptors in the ARC. **(A)** Image of fluorescence microscopy showing expression of TRPV1 receptors in the hypothalamus. **(B)** Image of bright-field microscopy showing the areas (a, b, and c) that were punched out for mRNA and western blot analysis. **(C)** Relative *Trpv1* mRNA expression around the ARC. Almost no expression of *Trpv1* mRNA was observed in the nearby nuclei of the ARC. Knockdown of the *Trpv1* gene in mice injected with *Trpv1* sgRNA in the ARC of POMC-Cre;;CRISPR/Cas9 mice. mRNA from 3 different mice was pooled for qPCR (WT, *n* = 15 mice, mice injected with control and *Trpv1* sgRNA, *n* = 6 mice, respectively). **(D)** Image of western blotting showing no expression of TRPV1 receptors around the ARC and knockdown of the *Trpv1* gene in the ARC. ARC, arcuate nucleus of the hypothalamus; Cre, Cre recombinase; CRISPR/Cas9, clustered regularly interspaced short palindromic repeats/CRISPR-associated protein 9; POMC, proopiomelanocortin; qPCR, quantitative polymerase chain reaction; sgRNA, single guide RNA; TRPV1, transient receptor potential vanilloid 1 receptor; WT, wild-type.
(PDF)

S5 Fig Lack of TRPV1 receptor expression in NPY neurons in the ARC. **(A)** Images of fluorescence confocal microscopy showing no expression of TRPV1 receptors in NPY neurons. Scale bar: 20 μm. **(B** and **C)** Representative recording sample showing no response of NPY neurons to RTX (100 nM). Scale bar: 25 mV, 1 min. Pooled data of the mean membrane potential from 10 NPY-GFP neurons (C, Vm, Control, −60.8 ± 0.7 mV; RTX, −63.0 ± 1.6 mV, *n* = 10 neurons, *p* > 0.05). All data are shown as mean ± SEM. **(D** and **E)** Recording sample showing that raising the temperature did not change the membrane potential of NPY-GFP neurons. Pooled data from 5 NYP-GFP neurons (E). Scale bar: 20 mV, 10 s. ARC, arcuate nucleus of the hypothalamus; GFP, green fluorescent protein; NPY, neuropeptide Y; RTX, resiniferatoxin; TRPV1, transient receptor potential vanilloid 1 receptor.
(PDF)

S6 Fig TRPV1 receptor expression in POMC neurons in the ARC. **(A** and **B)** Representative trace showing response of POMC neurons to raising the temperature from 34 °C to 38 °C. Knockdown of the *Trpv1* gene in POMC neurons decreased the mean amplitude of temperature-sensitive inward currents (temperature from 34°C to 38°C; Control, −15.7 ± 3.1 pA, *n* = 11 neurons; *Trpv1* sgRNA, −6.1 ± 1.4 pA, *n* = 6 neurons, *p* < 0.05). Holding potential of −70 mV, scale bar: 50 pA, 50 s. **(C)** Temperature-mediated depolarization of POMC neuron shown in Fig 1F was completely reversible when the temperature went down. Temperature-sensitive POMC neurons also responded to the TRPV1 receptor agonist RTX (100 nM). Scale bar: 50 mV, 5 min. **(D** and **E)** Temperature-insensitive POMC neurons did not respond to RTX. Pooled data of mean membrane potential of temperature-insensitive POMC neurons at 34 °C and 38 °C (*n* = 7 neurons). Scale bar: 50 mV, 5 min. ARC, arcuate nucleus of the hypothalamus; POMC, proopiomelanocortin; RTX, resiniferatoxin; sgRNA, single guide RNA; TRPV1, transient receptor potential vanilloid 1 receptor.
(PDF)

S7 Fig Altered thermal sensitivity of POMC neurons following knockdown of the *Trpv3* and *Trpv4* genes in the ARC. **(A)** Representative trace of whole cell patch-clamp recording of POMC neurons from mice injected with *Trpv3* and *Trpv4* shRNA into the ARC. Scale Bar, 20 mV, 2 min. **(B)** Pooled data from 7 POMC neurons from mice injected with *Trpv3* and *Trpv4* shRNA into the ARC. In contrast to the findings in control mice (Fig 1G), POMC neurons were less sensitive to increased temperature under these experimental conditions. *p < 0.05. ARC, arcuate nucleus of the hypothalamus; POMC, proopiomelanocortin; shRNA, short hairpin RNA; *Trpv3*, transient receptor potential vanilloid 3 receptor, *Trpv4*, transient receptor potential vanilloid 4 receptor.
(PDF)

S8 Fig Nal does not block the effect of capsaicin. Pooled data from 11 mice showing the effect of capsaicin in the presence of the opioid receptor antagonist Nal (1 mM). **p < 0.01, ***p < 0.001. Nal, naloxone.
(PDF)

S1 Movie Movie showing a mouse running on a treadmill, while measuring ARC temperature. ARC, arcuate nucleus of the hypothalamus.
(MP4)

S1 Data All individual numerical values that underlie the summary data shown in the following figures: Figs 1G, 1H, 2C, 2D, 2F, 2G, 2H, 3B, 3C, 3D, 3E, 4D, 4F, 5D, 6B, 6C, 6D, 6E, 6F and 6G, S1B, S1D, S1F, S1H, S4C, S5C, S5E, S6B, S6E, S7B and S8 Figs. (XLSX)

## Abbreviations

AAV1: adeno-associated virus serotype 1
AAV5: adeno-associated virus serotype 5
aCSF: artificial cerebrospinal fluid
AgRP: agouti-related peptide
ARC: arcuate nucleus of the hypothalamus
CB1: cannabinoid type 1 receptor
ChR2: channelrhodopsin-2
CNO: clozapine-N-oxide
CNQX: 6-cyano-7-nitroquinoxaline-2,3-dione
Cre: Cre recombinase
CRISPR/Cas-9: clustered regularly interspaced short palindromic repeats/CRISPR-associated protein 9
DIO: double-floxed inversed open reading frame
DREADD: designer receptor exclusively activated by designer drugs
eGFP: enhanced green fluorescent protein
Ex: exercise
eYFP: enhanced YFP
*Gad2*: glutamate decarboxylase 2
HEK293 cells: human embryonic kidney 293 cells
hSyn: human synapsin I promoter
i.p.: Intraperitoneal
KO: knock-out
MC3/4R: melanocortin 3 and 4 receptor
mCherry: monomeric cherry fluorescent protein
mRNA: messenger RNA
nPE: neuronal POMC enhancer
NPY: neuropeptide Y
POMC: proopiomelanocortin
qPCR: quantitative polymerase chain reaction
RT-PCR: reverse transcription-polymerase chain reaction
RTX: resiniferatoxin
sgRNA: single guide RNA
shRNA: short hairpin RNA
TRPV: transient receptor potential vanilloid
TRPV1: transient receptor potential vanilloid 1 receptor
TTX: tetrodotoxin
Veh: vehicle
WTA: whole-transcriptome amplification
YFP: yellow fluorescent protein
α-MSH: α-melanocyte-stimulating hormone

## References

[1] Baker FC, Waner JI, Vieira EF, Taylor SR, Driver HS, Mitchell D. Sleep and 24 hour body temperatures: a comparison in young men, naturally cycling women and women taking hormonal contraceptives. J Physiol. 2001;530(Pt 3):565–74. doi: 10.1111/j.1469-7793.2001.0565k.x . 11158285 10.1111/j.1469-7793.2001.0565k.x PMC2278431

[2] Schlader ZJ, Stannard SR, Mundel T. Human thermoregulatory behavior during rest and exercise—a prospective review. Physiol Behav. 2010;99(3):269–75. doi: 10.1016/j.physbeh.2009.12.003 . 20006632 10.1016/j.physbeh.2009.12.003

[3] Hibi M, Oishi S, Matsushita M, Yoneshiro T, Yamaguchi T, Usui C, et al Brown adipose tissue is involved in diet-induced thermogenesis and whole-body fat utilization in healthy humans. Int J Obes (Lond). 2016;40(11):1655–61. doi: 10.1038/ijo.2016.124 Katsuragi are employees of the Kao Corporation. The other authors have no personal or financial conflicts of interest. 27430878 10.1038/ijo.2016.124 PMC5116053

[4] Caterina MJ, Schumacher MA, Tominaga M, Rosen TA, Levine JD, Julius D. The capsaicin receptor: a heat-activated ion channel in the pain pathway. Nature. 1997;389(6653):816–24. doi: 10.1038/39807 . 9349813 10.1038/39807

[5] Smith GD, Gunthorpe MJ, Kelsell RE, Hayes PD, Reilly P, Facer P, et al TRPV3 is a temperature-sensitive vanilloid receptor-like protein. Nature. 2002;418(6894):186–90. doi: 10.1038/nature00894 . 12077606 10.1038/nature00894

[6] Xu H, Ramsey IS, Kotecha SA, Moran MM, Chong JA, Lawson D, et al TRPV3 is a calcium-permeable temperature-sensitive cation channel. Nature. 2002;418(6894):181–6. doi: 10.1038/nature00882 . 12077604 10.1038/nature00882

[7] Patapoutian A, Peier AM, Story GM, Viswanath V. ThermoTRP channels and beyond: mechanisms of temperature sensation. Nat Rev Neurosci. 2003;4(7):529–39. doi: 10.1038/nrn1141 . 12838328 10.1038/nrn1141

[8] Ahluwalia J, Rang H, Nagy I. The putative role of vanilloid receptor-like protein-1 in mediating high threshold noxious heat-sensitivity in rat cultured primary sensory neurons. Eur J Neurosci. 2002;16(8):1483–9. . 12405961 10.1046/j.1460-9568.2002.02231.x

[9] Todaka H, Taniguchi J, Satoh J, Mizuno A, Suzuki M. Warm temperature-sensitive transient receptor potential vanilloid 4 (TRPV4) plays an essential role in thermal hyperalgesia. J Biol Chem. 2004;279(34):35133–8. doi: 10.1074/jbc.M406260200 . 15187078 10.1074/jbc.M406260200

[10] Nakayama T, Eisenman JS, Hardy JD. Single unit activity of anterior hypothalamus during local heating. Science. 1961;134(3478):560–1. . 13727681 10.1126/science.134.3478.560

[11] Mezey E, Toth ZE, Cortright DN, Arzubi MK, Krause JE, Elde R, et al Distribution of mRNA for vanilloid receptor subtype 1 (VR1), and VR1-like immunoreactivity, in the central nervous system of the rat and human. Proc Natl Acad Sci U S A. 2000;97(7):3655–60. doi: 10.1073/pnas.060496197 . 10725386 10.1073/pnas.060496197 PMC16295

[12] Cavanaugh DJ, Chesler AT, Jackson AC, Sigal YM, Yamanaka H, Grant R, et al Trpv1 reporter mice reveal highly restricted brain distribution and functional expression in arteriolar smooth muscle cells. J Neurosci. 2011;31(13):5067–77. doi: 10.1523/JNEUROSCI.6451-10.2011 . 21451044 10.1523/JNEUROSCI.6451-10.2011 PMC3087977

[13] Sudbury JR, Bourque CW. Dynamic and permissive roles of TRPV1 and TRPV4 channels for thermosensation in mouse supraoptic magnocellular neurosecretory neurons. J Neurosci. 2013;33(43):17160–5. doi: 10.1523/JNEUROSCI.1048-13.2013 . 24155319 10.1523/JNEUROSCI.1048-13.2013 PMC6618445

[14] Roberts JC, Davis JB, Benham CD. [3H]Resiniferatoxin autoradiography in the CNS of wild-type and TRPV1 null mice defines TRPV1 (VR-1) protein distribution. Brain Res. 2004;995(2):176–83. . 14672807 10.1016/j.brainres.2003.10.001

[15] Sharif Naeini R, Witty MF, Seguela P, Bourque CW. An N-terminal variant of Trpv1 channel is required for osmosensory transduction. Nat Neurosci. 2006;9(1):93–8. doi: 10.1038/nn1614 . 16327782 10.1038/nn1614

[16] Sharif-Naeini R, Ciura S, Bourque CW. TRPV1 gene required for thermosensory transduction and anticipatory secretion from vasopressin neurons during hyperthermia. Neuron. 2008;58(2):179–85. doi: 10.1016/j.neuron.2008.02.013 . 18439403 10.1016/j.neuron.2008.02.013

[17] Henry FE, Sugino K, Tozer A, Branco T, Sternson SM. Cell type-specific transcriptomics of hypothalamic energy-sensing neuron responses to weight-loss. Elife. 2015;4 doi: 10.7554/eLife.09800 . 26329458 10.7554/eLife.09800 PMC4595745

[18] Acuna-Goycolea C, van den Pol AN. Neuroendocrine proopiomelanocortin neurons are excited by hypocretin/orexin. J Neurosci. 2009;29(5):1503–13. doi: 10.1523/JNEUROSCI.5147-08.2009 . 19193897 10.1523/JNEUROSCI.5147-08.2009 PMC2751610

[19] Lee DK, Jeong JH, Chun SK, Chua S Jr., Jo YH. Interplay between glucose and leptin signalling determines the strength of GABAergic synapses at POMC neurons. Nat Commun. 2015;6:6618 doi: 10.1038/ncomms7618 . 25808323 10.1038/ncomms7618 PMC4375782

[20] Jordt SE, Julius D. Molecular basis for species-specific sensitivity to "hot" chili peppers. Cell. 2002;108(3):421–30. . 11853675 10.1016/s0092-8674(02)00637-2

[21] Yang F, Xiao X, Cheng W, Yang W, Yu P, Song Z, et al Structural mechanism underlying capsaicin binding and activation of the TRPV1 ion channel. Nat Chem Biol. 2015;11(7):518–24. doi: 10.1038/nchembio.1835 . 26053297 10.1038/nchembio.1835 PMC4472570

[22] Yang F, Zheng J. Understand spiciness: mechanism of TRPV1 channel activation by capsaicin. Protein Cell. 2017;8(3):169–77. doi: 10.1007/s13238-016-0353-7 . 28044278 10.1007/s13238-016-0353-7 PMC5326624

[23] Cheng W, Yang F, Liu S, Colton CK, Wang C, Cui Y, et al Heteromeric heat-sensitive transient receptor potential channels exhibit distinct temperature and chemical response. J Biol Chem. 2012;287(10):7279–88. doi: 10.1074/jbc.M111.305045 . 22184123 10.1074/jbc.M111.305045 PMC3293533

[24] Caterina MJ, Julius D. The vanilloid receptor: a molecular gateway to the pain pathway. Annu Rev Neurosci. 2001;24:487–517. doi: 10.1146/annurev.neuro.24.1.487 . 11283319 10.1146/annurev.neuro.24.1.487

[25] Kauer JA, Gibson HE. Hot flash: TRPV channels in the brain. Trends Neurosci. 2009;32(4):215–24. doi: 10.1016/j.tins.2008.12.006 . 19285736 10.1016/j.tins.2008.12.006

[26] Vriens J, Appendino G, Nilius B. Pharmacology of vanilloid transient receptor potential cation channels. Mol Pharmacol. 2009;75(6):1262–79. doi: 10.1124/mol.109.055624 . 19297520 10.1124/mol.109.055624

[27] Raisinghani M, Pabbidi RM, Premkumar LS. Activation of transient receptor potential vanilloid 1 (TRPV1) by resiniferatoxin. J Physiol. 2005;567(Pt 3):771–86. doi: 10.1113/jphysiol.2005.087874 . 16037081 10.1113/jphysiol.2005.087874 PMC1474234

[28] Gavva NR, Tamir R, Qu Y, Klionsky L, Zhang TJ, Immke D, et al AMG 9810 [(E)-3-(4-t-butylphenyl)-N-(2,3-dihydrobenzo[b][1,4] dioxin-6-yl)acrylamide], a novel vanilloid receptor 1 (TRPV1) antagonist with antihyperalgesic properties. J Pharmacol Exp Ther. 2005;313(1):474–84. doi: 10.1124/jpet.104.079855 . 15615864 10.1124/jpet.104.079855

[29] Cheng W, Yang F, Takanishi CL, Zheng J. Thermosensitive TRPV channel subunits coassemble into heteromeric channels with intermediate conductance and gating properties. J Gen Physiol. 2007;129(3):191–207. doi: 10.1085/jgp.200709731 . 17325193 10.1085/jgp.200709731 PMC2151614

[30] Lam DD, Attard CA, Mercer AJ, Myers MG Jr., Rubinstein M, Low MJ. Conditional expression of Pomc in the Lepr-positive subpopulation of POMC neurons is sufficient for normal energy homeostasis and metabolism. Endocrinology. 2015;156(4):1292–302. doi: 10.1210/en.2014-1373 . 25594696 10.1210/en.2014-1373 PMC4399319

[31] Koch M, Varela L, Kim JG, Kim JD, Hernandez-Nuno F, Simonds SE, et al Hypothalamic POMC neurons promote cannabinoid-induced feeding. Nature. 2015;519(7541):45–50. doi: 10.1038/nature14260 . 25707796 10.1038/nature14260 PMC4496586

[32] Bumaschny VF, Yamashita M, Casas-Cordero R, Otero-Corchon V, de Souza FS, Rubinstein M, et al Obesity-programmed mice are rescued by early genetic intervention. J Clin Invest. 2012;122(11):4203–12. doi: 10.1172/JCI62543 . 23093774 10.1172/JCI62543 PMC3484438

[33] Plum L, Ma X, Hampel B, Balthasar N, Coppari R, Munzberg H, et al Enhanced PIP3 signaling in POMC neurons causes KATP channel activation and leads to diet-sensitive obesity. J Clin Invest. 2006;116(7):1886–901. doi: 10.1172/JCI27123 . 16794735 10.1172/JCI27123 PMC1481658

[34] Hill JW, Williams KW, Ye C, Luo J, Balthasar N, Coppari R, et al Acute effects of leptin require PI3K signaling in hypothalamic proopiomelanocortin neurons in mice. J Clin Invest. 2008;118(5):1796–805. doi: 10.1172/JCI32964 . 18382766 10.1172/JCI32964 PMC2276395

[35] Zhan C, Zhou J, Feng Q, Zhang JE, Lin S, Bao J, et al Acute and long-term suppression of feeding behavior by POMC neurons in the brainstem and hypothalamus, respectively. J Neurosci. 2013;33(8):3624–32. doi: 10.1523/JNEUROSCI.2742-12.2013 . 23426689 10.1523/JNEUROSCI.2742-12.2013 PMC6619547

[36] Aponte Y, Atasoy D, Sternson SM. AGRP neurons are sufficient to orchestrate feeding behavior rapidly and without training. Nat Neurosci. 2011;14(3):351–5. Epub 2011/01/07. doi: 10.1038/nn.2739 . 21209617 10.1038/nn.2739 PMC3049940

[37] Enriori PJ, Evans AE, Sinnayah P, Jobst EE, Tonelli-Lemos L, Billes SK, et al Diet-induced obesity causes severe but reversible leptin resistance in arcuate melanocortin neurons. Cell Metab. 2007;5(3):181–94. doi: 10.1016/j.cmet.2007.02.004 . 17339026 10.1016/j.cmet.2007.02.004

[38] Fenselau H, Campbell JN, Verstegen AM, Madara JC, Xu J, Shah BP, et al A rapidly acting glutamatergic ARC—>PVH satiety circuit postsynaptically regulated by alpha-MSH. Nat Neurosci. 2017;20(1):42–51. doi: 10.1038/nn.4442 . 27869800 10.1038/nn.4442 PMC5191921

[39] de Souza FS, Santangelo AM, Bumaschny V, Avale ME, Smart JL, Low MJ, et al Identification of neuronal enhancers of the proopiomelanocortin gene by transgenic mouse analysis and phylogenetic footprinting. Mol Cell Biol. 2005;25(8):3076–86. doi: 10.1128/MCB.25.8.3076-3086.2005 . 15798195 10.1128/MCB.25.8.3076-3086.2005 PMC1069613

[40] Fonseca CG, Pires W, Lima MR, Guimaraes JB, Lima NR, Wanner SP. Hypothalamic temperature of rats subjected to treadmill running in a cold environment. PLoS ONE. 2014;9(11):e111501 doi: 10.1371/journal.pone.0111501 . 25365556 10.1371/journal.pone.0111501 PMC4218756

[41] Billeter AT, Galbraith N, Walker S, Lawson C, Gardner SA, Sarojini H, et al TRPA1 mediates the effects of hypothermia on the monocyte inflammatory response. Surgery. 2015;158(3):646–54. doi: 10.1016/j.surg.2015.03.065 . 26054320 10.1016/j.surg.2015.03.065

[42] Zheng J. Molecular mechanism of TRP channels. Compr Physiol. 2013;3(1):221–42. doi: 10.1002/cphy.c120001 . 23720286 10.1002/cphy.c120001 PMC3775668

[43] Guler AD, Lee H, Iida T, Shimizu I, Tominaga M, Caterina M. Heat-evoked activation of the ion channel, TRPV4. J Neurosci. 2002;22(15):6408–14. . 12151520 10.1523/JNEUROSCI.22-15-06408.2002 PMC6758176

[44] Hellwig N, Albrecht N, Harteneck C, Schultz G, Schaefer M. Homo- and heteromeric assembly of TRPV channel subunits. J Cell Sci. 2005;118(Pt 5):917–28. doi: 10.1242/jcs.01675 . 15713749 10.1242/jcs.01675

[45] Nedungadi TP, Carreno FR, Walch JD, Bathina CS, Cunningham JT. Region-specific changes in transient receptor potential vanilloid channel expression in the vasopressin magnocellular system in hepatic cirrhosis-induced hyponatraemia. J Neuroendocrinol. 2012;24(4):642–52. doi: 10.1111/j.1365-2826.2011.02273.x . 22188460 10.1111/j.1365-2826.2011.02273.x PMC3314151

[46] Anderson EJ, Cakir I, Carrington SJ, Cone RD, Ghamari-Langroudi M, Gillyard T, et al 60 YEARS OF POMC: Regulation of feeding and energy homeostasis by alpha-MSH. J Mol Endocrinol. 2016;56(4):T157–74. doi: 10.1530/JME-16-0014 . 26939593 10.1530/JME-16-0014 PMC5027135

[47] Jeong JH, Woo YJ, Chua S Jr., Jo YH. Single-Cell Gene Expression Analysis of Cholinergic Neurons in the Arcuate Nucleus of the Hypothalamus. PLoS ONE. 2016;11(9):e0162839 doi: 10.1371/journal.pone.0162839 . 27611685 10.1371/journal.pone.0162839 PMC5017726

[48] Elias CF, Aschkenasi C, Lee C, Kelly J, Ahima RS, Bjorbaek C, et al Leptin differentially regulates NPY and POMC neurons projecting to the lateral hypothalamic area. Neuron. 1999;23(4):775–86. . 10482243 10.1016/s0896-6273(01)80035-0

[49] Elias CF, Lee C, Kelly J, Aschkenasi C, Ahima RS, Couceyro PR, et al Leptin activates hypothalamic CART neurons projecting to the spinal cord. Neuron. 1998;21(6):1375–85. . 9883730 10.1016/s0896-6273(00)80656-x

[50] King CM, Hentges ST. Relative number and distribution of murine hypothalamic proopiomelanocortin neurons innervating distinct target sites. PLoS ONE. 2011;6(10):e25864 doi: 10.1371/journal.pone.0025864 . 21991375 10.1371/journal.pone.0025864 PMC3186811

[51] Hentges ST, Otero-Corchon V, Pennock RL, King CM, Low MJ. Proopiomelanocortin expression in both GABA and glutamate neurons. J Neurosci. 2009;29(43):13684–90. doi: 10.1523/JNEUROSCI.3770-09.2009 . 19864580 10.1523/JNEUROSCI.3770-09.2009 PMC2785088

[52] Zhang LL, Yan Liu D, Ma LQ, Luo ZD, Cao TB, Zhong J, et al Activation of transient receptor potential vanilloid type-1 channel prevents adipogenesis and obesity. Circ Res. 2007;100(7):1063–70. doi: 10.1161/01.RES.0000262653.84850.8b . 17347480 10.1161/01.RES.0000262653.84850.8b

[53] Whiting S, Derbyshire E, Tiwari BK. Capsaicinoids and capsinoids. A potential role for weight management? A systematic review of the evidence. Appetite. 2012;59(2):341–8. doi: 10.1016/j.appet.2012.05.015 . 22634197 10.1016/j.appet.2012.05.015

[54] Lee E, Jung DY, Kim JH, Patel PR, Hu X, Lee Y, et al Transient receptor potential vanilloid type-1 channel regulates diet-induced obesity, insulin resistance, and leptin resistance. FASEB J. 2015 doi: 10.1096/fj.14-268300 . 25888600 10.1096/fj.14-268300 PMC4511197

[55] Rampone AJ, Shirasu ME. Temperature Changes in the Rat in Response to Feeding. Science. 1964;144(3616):317–9. . 14169727 10.1126/science.144.3616.317

[56] Janssens PL, Hursel R, Westerterp-Plantenga MS. Capsaicin increases sensation of fullness in energy balance, and decreases desire to eat after dinner in negative energy balance. Appetite. 2014;77:44–9. doi: 10.1016/j.appet.2014.02.018 . 24630935 10.1016/j.appet.2014.02.018

