# Supplementary material for: Activation of temperature-sensitive TRPV1-like receptors in ARC POMC neurons reduces food intake
Source: PLoS Biol. 2018 Apr 24;16(4):e2004399. doi: 10.1371/journal.pbio.2004399 (PMC5915833; doi:10.1371/journal.pbio.2004399)
Supplement: S1 Table — (DOCX) [file pbio.2004399.s001.docx]

|  |  | **Food intake (g)** | | |  |
| --- | --- | --- | --- | --- | --- |
| **Groups** | | **4 hrs** | **8 hrs** | **12 hrs** | **n** |
| Control shRNA + Sal | | 1.6 ± 0.04 | 2.4 ± 0.1 | 4.1 ± 0.1 | 9 |
| Control ShRNA + Cap | | 0.8 ± 0.1*** | 1.5 ± 0.2** | 2.3 ± 0.2*** | 9 |
|  |  |  |  |  |  |
| *Trpv1* shRNA + Sal | | 1.6 ± 0.1 | 2.8 ± 0.1 | 4.3 ± 0.2 | 9 |
| *Trpv1* shRNA + Cap | | 1.4 ± 0.04 | 2.6 ± 0.2 | 3.9 ± 0.2 | 9 |
|  |  |  |  |  |  |
| Sal + i.p. Sal | | 1.9 ± 0.1 | 2.9 ± 0.1 | 4.7 ± 0.1 | 8 |
| Cap + i.p. Sal | | 1.3 ± 0.1** | 2.2 ± 0.1*** | 3.5 ± 0.2*** | 8 |
|  |  |  |  |  |  |
| Sal + i.p. CNO | | 1.8 ± 0.1 | 3.0 ± 0.2 | 4.8 ± 0.2 | 8 |
| Cap + i.p. CNO | | 1.6 ± 0.1 | 2.9 ± 0.1 | 4.5 ± 0.2 | 8 |
|  |  |  |  |  |  |
| Vehicle + Sal | | 1.8 ± 0.1 | 2.3 ± 0.1 | 4.1 ± 0.2 | 11 |
| SHU9119 + Sal | | 1.6 ± 0.1 | 2.5 ± 0.1 | 4.1 ± 0.2 | 11 |
| SHU9119 + Cap | | 1.6 ± 0.1 | 2.4 ± 0.1 | 3.9 ± 0.1 | 11 |
|  |  |  |  |  |  |
| **p < 0.01, ***p < 0.001 | | | | | |
