# Supplementary material for: Activation of temperature-sensitive TRPV1-like receptors in ARC POMC neurons reduces food intake
Source: PLoS Biol. 2018 Apr 24;16(4):e2004399. doi: 10.1371/journal.pbio.2004399 (PMC5915833; doi:10.1371/journal.pbio.2004399)
Supplement: S2 Table — (DOCX) [file pbio.2004399.s002.docx]

|  |  | **Liquid food intake (ml)** | | | | | |  |
| --- | --- | --- | --- | --- | --- | --- | --- | --- |
| **Groups** | | **10** | **20** | **30** | **40** | **50** | **60 (min)** | **n** |
| Vehicle | | 0.6 ± 0.1 | 1.0 ± 0.1 | 1.5 ± 0.1 | 1.8 ± 0.1 | 2.1 ± 0.2 | 2.4 ± 0.2 | 9 |
| Vehicle + Ex | | 0.4 ± 0.05* | 0.7 ± 0.1* | 0.8 ± 0.1*** | 1.1 ± 0.1*** | 1.2 ± 0.1*** | 1.5 ± 0.1*** | 9 |
|  |  |  |  |  |  |  |  |  |
| SHU9119 | | 0.8 ± 0.04 | 1.3 ± 0.1 | 1.5 ± 0.1 | 1.8 ± 0.1 | 2.0 ± 0.1 | 2.3 ± 0.1 | 9 |
| SHU9119 + Ex | | 0.9 ± 0.1 | 1.1 ± 0.1 | 1.4 ± 0.1 | 1.6 ± 0.1 | 1.8 ± 0.1 | 2.3 ± 0.1 | 9 |
|  |  |  |  |  |  |  |  |  |
| Control sgRNA | | 0.7 ± 0.04 | 1.1 ± 0.1 | 1.5 ± 0.04 | 1.7 ± 0.04 | 1.9 ± 0.1 | 2.2 ± 0.1 | 9 |
| Control sgRNA + Ex | | 0.4 ± 0.1** | 0.7 ± 0.1* | 1.0 ± 0.1*** | 1.1 ± 0.1*** | 1.4 ± 0.2** | 1.5 ± 0.1*** | 9 |
|  |  |  |  |  |  |  |  |  |
| *Trpv1* sgRNA | | 0.9 ± 0.1 | 1.3 ± 0.1 | 1.7 ± 0.1 | 2.0 ± 0.1 | 2.2 ± 0.1 | 2.5 ± 0.1 | 9 |
| *Trpv1* sgRNA + Ex | | 1.1 ± 0.1 | 1.4 ± 0.1 | 1.6 ± 0.1 | 1.9 ± 0.1 | 2.1 ± 0.1 | 2.4 ± 0.1 | 9 |
| *p < 0.05, **p < 0.01, ***p < 0.001 | | | | | | | | |
