# Supplementary figures and images for: Activation of temperature-sensitive TRPV1-like receptors in ARC POMC neurons reduces food intake
Source: PLoS Biol. 2018 Apr 24;16(4):e2004399. doi: 10.1371/journal.pbio.2004399 (PMC5915833; doi:10.1371/journal.pbio.2004399)

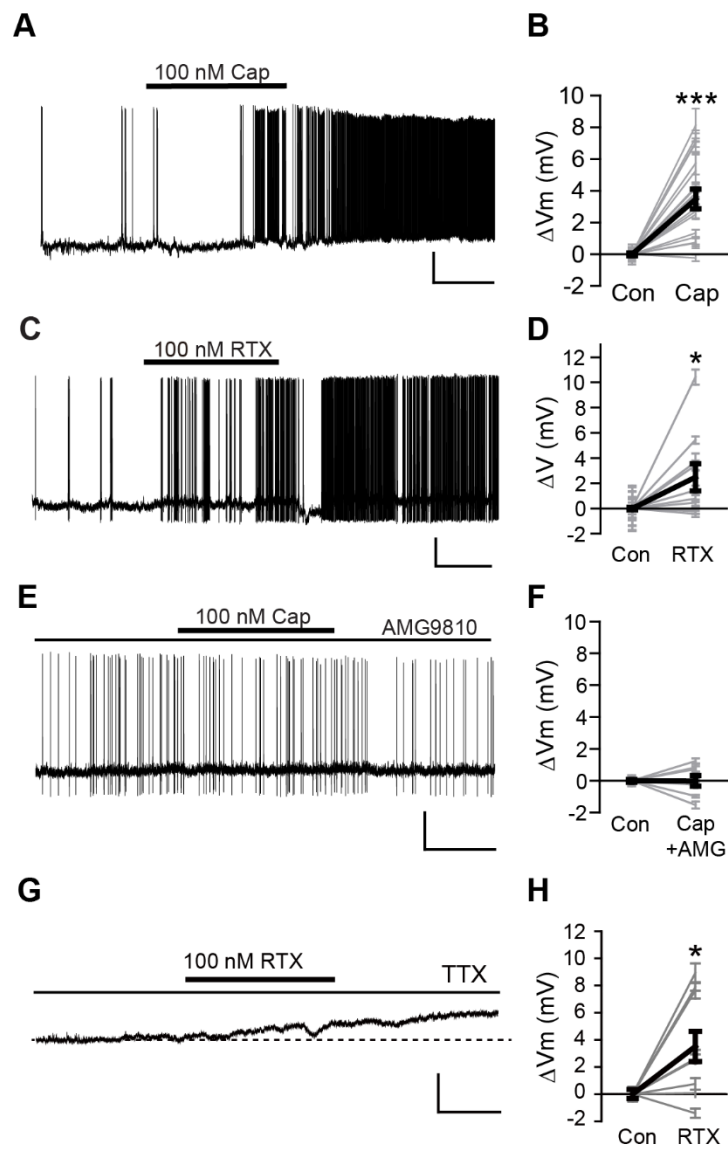

Supplement: S1 Fig — (A and B) Representative recording sample of the membrane potential before, during, and after treatment with the TRPV1 agonist. Treatment with capsaicin induced a depolarization of POMC neuron. Pooled data from 20 neurons of membrane potential of POMC neurons following application of Cap (100 nM, ΔVm, 3.8 ± 0.5 mV, ***p < 0.001). (C and D) Representative recording sample showing effect of RTX (C). (D) Pooled data of membrane potential of POMC neurons following application of RTX (ΔVm, 2.5 ± 1.1 mV, n = 10 neurons, *p < 0.05). (E and F) Recording sample showing the effect of the TRPV1 receptor antagonist AMG9810 (10 μM) (E). AMG9810 completely blocked the effect of capsaicin (n = 8 neurons, p > 0.05) (F). (G and H) Representative trace showing depolarization of POMC neurons by RTX in the presence of TTX (1 μM). Pooled data of membrane potential of POMC neurons following application of RTX (H) (n = 10 neurons, p < 0.05). All neurophysiological recordings were obtained from bright fluorescent green neurons present in POMC-eGFP mice in the presence of picrotoxin (100 μM) and CNQX (10 μM). Scale bar: 20 mV, 2 min. Data are shown as mean ± SEM. ARC, arcuate nucleus of the hypothalamus; Cap, capsaicin; CNQX, 6-cyano-7-nitroquinoxaline-2,3-dione; eGFP, enhanced green fluorescent protein; POMC, proopiomelanocortin; RTX, resiniferatoxin; TRPV1, transient receptor potential vanilloid 1 receptor; TTX, tetrodotoxin. (PDF) [file pbio.2004399.s003.pdf]

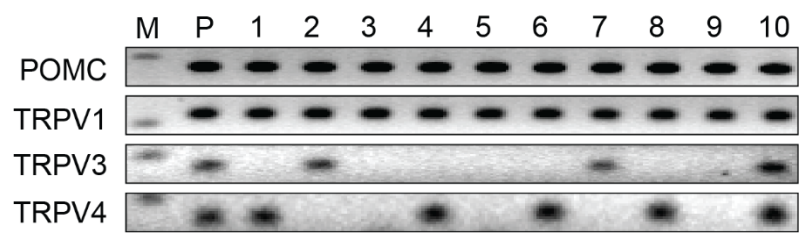

Supplement: S2 Fig — ARC, arcuate nucleus of the hypothalamus; lane, cell number; M, PCR marker; P, positive control; POMC, proopiomelanocortin; Trpv1, 3, 4, transient receptor potential vanilloid 1, 3, 4 receptors. (PDF) [file pbio.2004399.s004.pdf]

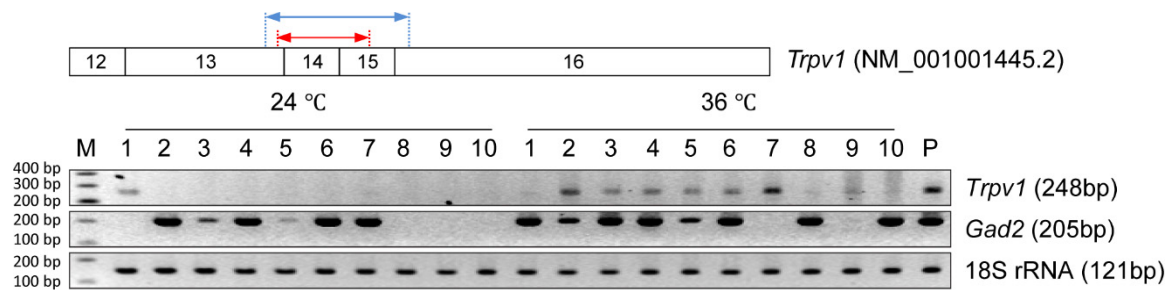

Supplement: S3 Fig — Upper panel: simplified structure of the mRNA (numbered boxes show exons of the Trpv1 gene) encoding Trpv1. Arrows show mRNA regions detected by primer sets (outer, blue; inner, red). Gel images show lack of Trpv1 mRNA in ARC POMC neurons from slices kept at 24 °C. M: PCR marker (Trpv1, 248 bp; Gad2, 205 bp; 18S rRNA, 121 bp). P: positive control single-cell samples were collected from hypothalamic slices kept at 24 °C and 36 °C for 3 h. The initial RT reaction was conducted after pressure ejection of the single-cell samples into freshly prepared RT mix A solution (20 U of RNase OUT, 300 ng of random primers, 0.5% NP-40, and RNase free water). Samples were sonicated in a total volume of 10 μL at 4 °C for 5 min and then incubated for 3 min at 65 °C before addition of 10 μL RT mix B (500 μm dNTP, 1 × RT buffer, 5 mm MgCl2, 10 mM DTT, and 200 U of Superscript IIl). The tubes were incubated at 25 °C for 5 min, at 42 °C for 1 h, and at 65 °C for 10 min. Three rounds of amplification were done for the detection of Trpv1 transcripts (two rounds with the outer primer set and one round with the inner primer set) and two rounds of amplification for the analysis of Gad2 transcripts. The primers used for qPCR were the following: Trpv1 outer primer, f5′-catgctcattgctctcatgg-3′ and r5′- aaccagggcaaagttcttcc-3′, Trpv1 inner primer, f5′-catgggcgagactgtcaac-3′ and r5′- ctgggtcctcgttgatgatg-3′; Gad2 outer primer, f5′-ggcgatggaatcttttctcct-3′, and Gad2 inner primer f5′-cgcactctggaagacaatga-3′ and r5′-cgaggcgttcgatttcttcaa-3′. ARC, arcuate nucleus of the hypothalamus; dNTP, deoxynucleotide; DTT, dithiothreitol; Gad2, glutamate decarboxylase 2; MgCl2, magnesium chloride; POMC, proopiomelanocortin; RT, reverse transcription; Trpv1, transient receptor potential vanilloid 1 receptor; U, unit. (PDF) [file pbio.2004399.s005.pdf]

A

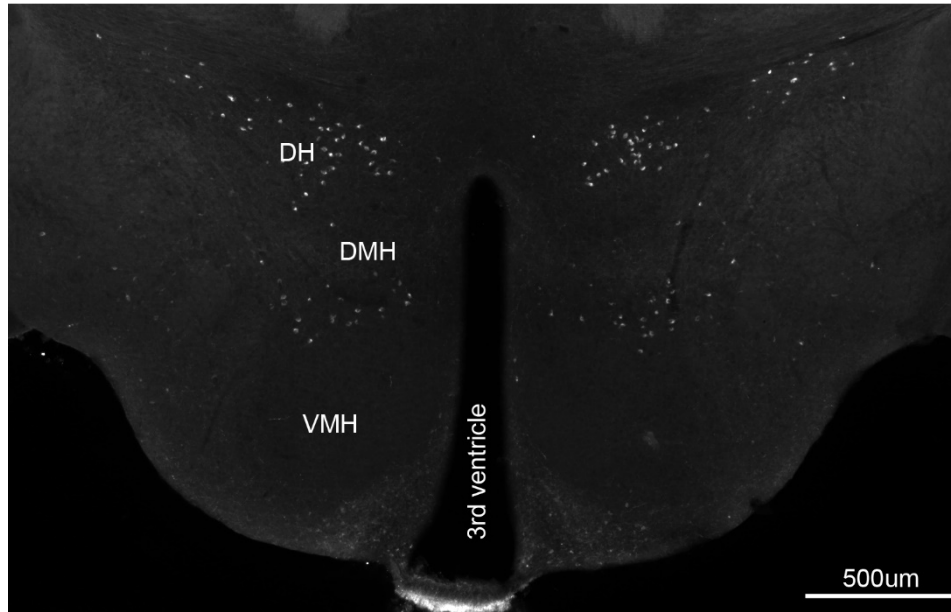

B

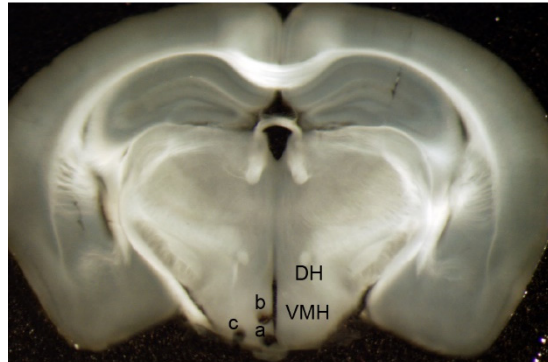

C

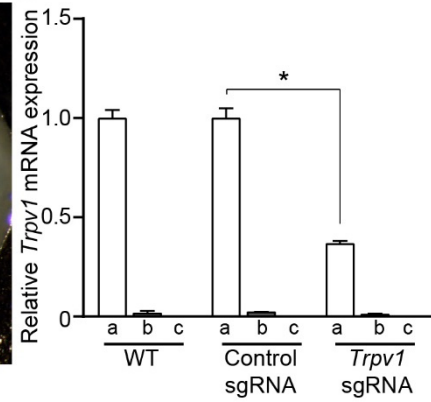

D

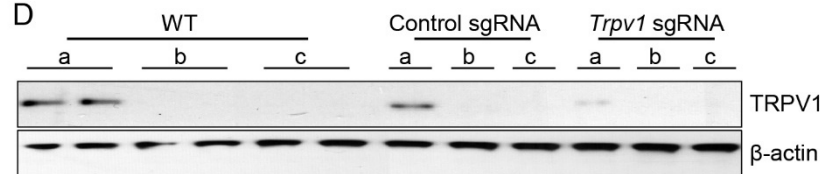

Supplement: S4 Fig — (A) Image of fluorescence microscopy showing expression of TRPV1 receptors in the hypothalamus. (B) Image of bright-field microscopy showing the areas (a, b, and c) that were punched out for mRNA and western blot analysis. (C) Relative Trpv1 mRNA expression around the ARC. Almost no expression of Trpv1 mRNA was observed in the nearby nuclei of the ARC. Knockdown of the Trpv1 gene in mice injected with Trpv1 sgRNA in the ARC of POMC-Cre;;CRISPR/Cas9 mice. mRNA from 3 different mice was pooled for qPCR (WT, n = 15 mice, mice injected with control and Trpv1 sgRNA, n = 6 mice, respectively). (D) Image of western blotting showing no expression of TRPV1 receptors around the ARC and knockdown of the Trpv1 gene in the ARC. ARC, arcuate nucleus of the hypothalamus; Cre, Cre recombinase; CRISPR/Cas9, clustered regularly interspaced short palindromic repeats/CRISPR-associated protein 9; POMC, proopiomelanocortin; qPCR, quantitative polymerase chain reaction; sgRNA, single guide RNA; TRPV1, transient receptor potential vanilloid 1 receptor; WT, wild-type. (PDF) [file pbio.2004399.s006.pdf]

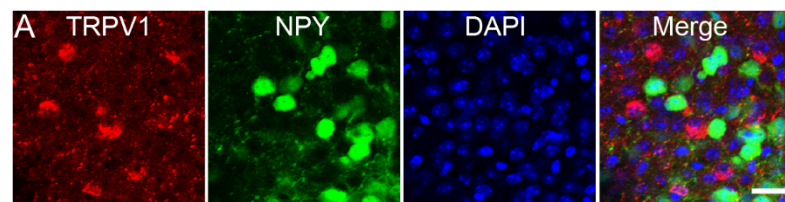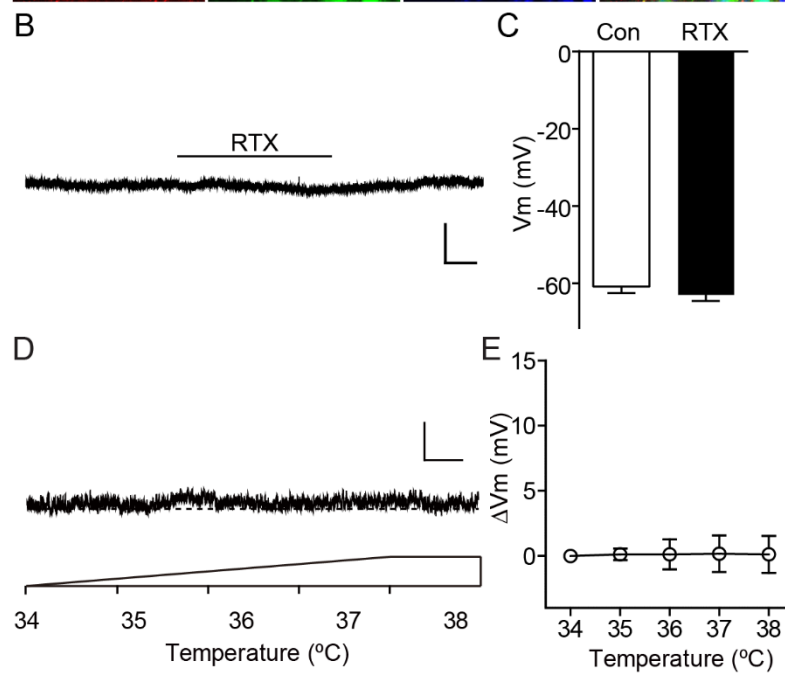

Supplement: S5 Fig — (A) Images of fluorescence confocal microscopy showing no expression of TRPV1 receptors in NPY neurons. Scale bar: 20 μm. (B and C) Representative recording sample showing no response of NPY neurons to RTX (100 nM). Scale bar: 25 mV, 1 min. Pooled data of the mean membrane potential from 10 NPY-GFP neurons (C, Vm, Control, −60.8 ± 0.7 mV; RTX, −63.0 ± 1.6 mV, n = 10 neurons, p > 0.05). All data are shown as mean ± SEM. (D and E) Recording sample showing that raising the temperature did not change the membrane potential of NPY-GFP neurons. Pooled data from 5 NYP-GFP neurons (E). Scale bar: 20 mV, 10 s. ARC, arcuate nucleus of the hypothalamus; GFP, green fluorescent protein; NPY, neuropeptide Y; RTX, resiniferatoxin; TRPV1, transient receptor potential vanilloid 1 receptor. (PDF) [file pbio.2004399.s007.pdf]

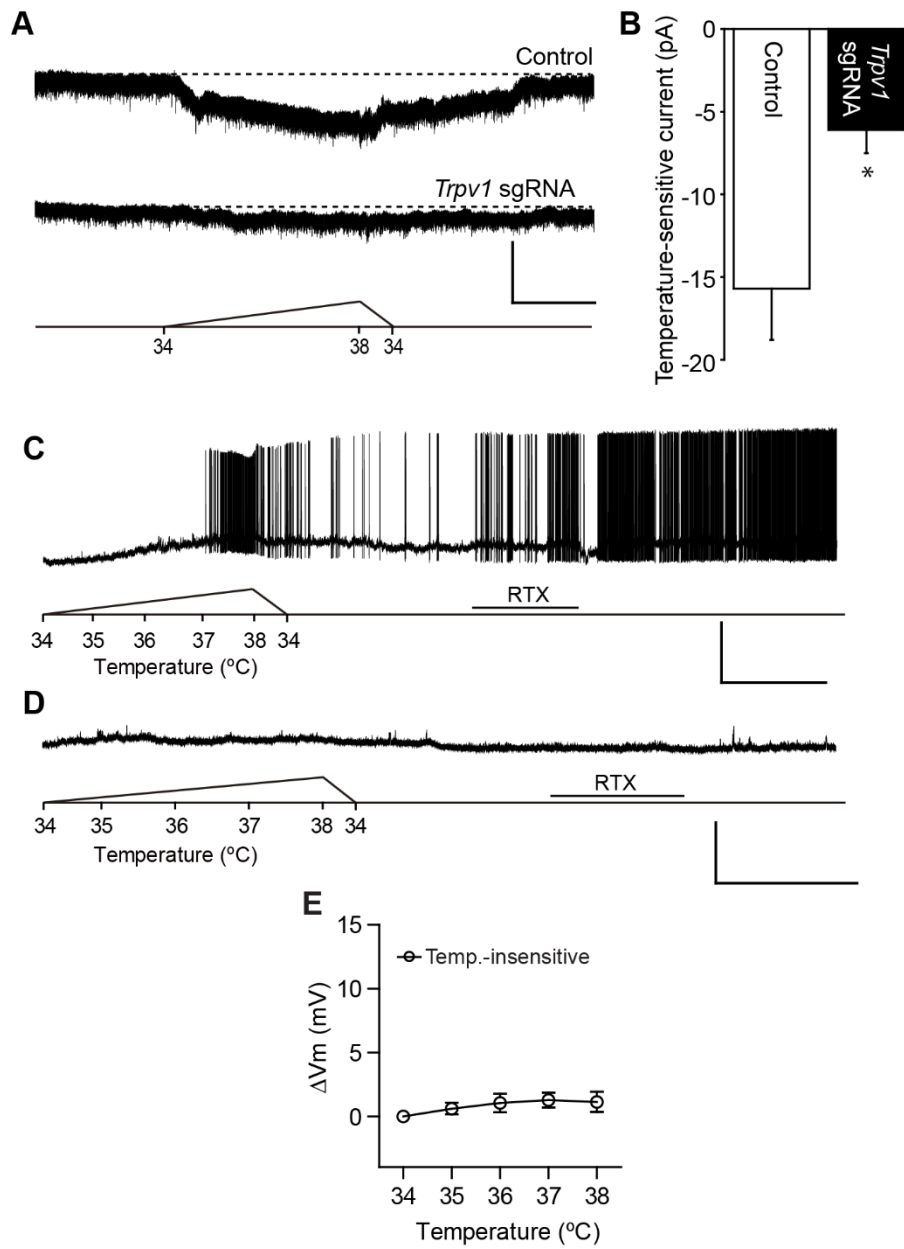

Supplement: S6 Fig — (A and B) Representative trace showing response of POMC neurons to raising the temperature from 34 °C to 38 °C. Knockdown of the Trpv1 gene in POMC neurons decreased the mean amplitude of temperature-sensitive inward currents (temperature from 34°C to 38°C; Control, −15.7 ± 3.1 pA, n = 11 neurons; Trpv1 sgRNA, −6.1 ± 1.4 pA, n = 6 neurons, p < 0.05). Holding potential of −70 mV, scale bar: 50 pA, 50 s. (C) Temperature-mediated depolarization of POMC neuron shown in Fig 1F was completely reversible when the temperature went down. Temperature-sensitive POMC neurons also responded to the TRPV1 receptor agonist RTX (100 nM). Scale bar: 50 mV, 5 min. (D and E) Temperature-insensitive POMC neurons did not respond to RTX. Pooled data of mean membrane potential of temperature-insensitive POMC neurons at 34 °C and 38 °C (n = 7 neurons). Scale bar: 50 mV, 5 min. ARC, arcuate nucleus of the hypothalamus; POMC, proopiomelanocortin; RTX, resiniferatoxin; sgRNA, single guide RNA; TRPV1, transient receptor potential vanilloid 1 receptor. (PDF) [file pbio.2004399.s008.pdf]

**A**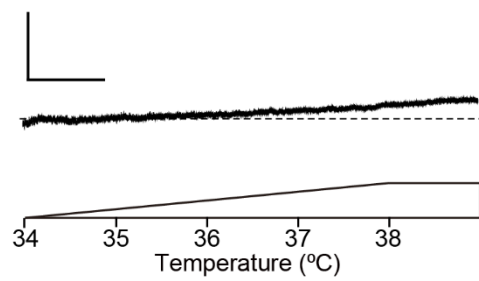**B**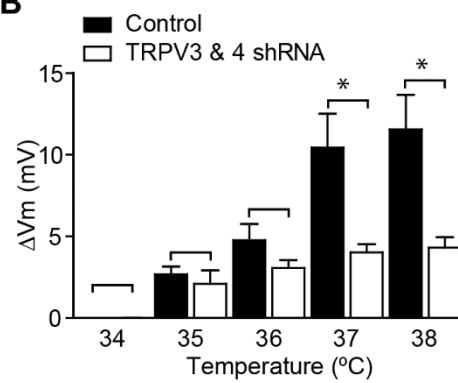

Supplement: S7 Fig — (A) Representative trace of whole cell patch-clamp recording of POMC neurons from mice injected with Trpv3 and Trpv4 shRNA into the ARC. Scale Bar, 20 mV, 2 min. (B) Pooled data from 7 POMC neurons from mice injected with Trpv3 and Trpv4 shRNA into the ARC. In contrast to the findings in control mice (Fig 1G), POMC neurons were less sensitive to increased temperature under these experimental conditions. *p < 0.05. ARC, arcuate nucleus of the hypothalamus; POMC, proopiomelanocortin; shRNA, short hairpin RNA; Trpv3, transient receptor potential vanilloid 3 receptor, Trpv4, transient receptor potential vanilloid 4 receptor. (PDF) [file pbio.2004399.s009.pdf]

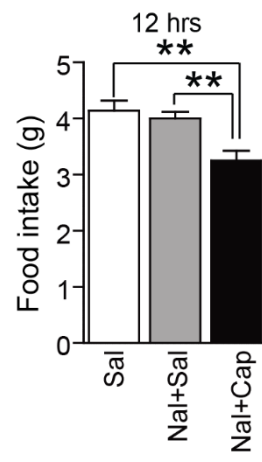

Supplement: S8 Fig — Pooled data from 11 mice showing the effect of capsaicin in the presence of the opioid receptor antagonist Nal (1 mM). **p < 0.01, ***p < 0.001. Nal, naloxone. (PDF) [file pbio.2004399.s010.pdf]
